# On identifying risk-adjusted efficiency gains or losses of prospective mergers and acquisitions

**DOI:** 10.1007/s10479-022-04826-w

**Published:** 2022-07-15

**Authors:** Mike G. Tsionas, Konstantinos N. Baltas

**Affiliations:** 1 Department of Financial, Legal and Accounting Management, Montpellier Business School, 2300 Avenue des Moulins, 34080 Montpellier, France; 2 Department of Economics, Management School, Lancaster University, Bailrigg, Lancaster, LA1 4XY UK; 3 Finance Group, Essex Business School, University of Essex, Wivenhoe Park, Colchester, CO4 3SQ UK

**Keywords:** Efficiency, M A, Stochastic frontier, Latent class

## Abstract

We propose a new approach to evaluate and compare ex-ante the risk-adjusted efficiency gains or losses of potential mergers and acquisitions (M &A). We test our methodology in the banking sector by estimating a latent class stochastic frontier model to account for the unobserved heterogeneity. We show that post-prospective M &A financial institutions can be better equipped to withstand potential adverse economic conditions. We highlight that similarities in strategic characteristics are vital in the creation of post-consolidation cost efficiency surplus. Our results are consistent after various robustness tests. Our findings have important policy implications in light of the challenges the traditional banking business model faces in the current digitalisation era.

## Introduction

The aftermath of the global financial crisis (GFC) has triggered a tremendous change in the financial services sector, causing a sizable build-upon a government debt in many industrialised countries. The recession leads to restructurings, a push toward lean management and a wave of M &A activities across a wide segment of industries. It created an even further urge to focus on core activities and capitalise from a differentiating position. Today’s cash-laden balance sheets and limited alternative for generating returns in other asset classes sparked even further the appetite for growth through M &A.

The industry with one of the highest ratio of consolidation activity as a result of the last financial turmoil, is the banking industry. The number of banks has declined considerably over the last years mainly due to failures during periods of crisis and it is projected that it will decline more in the post COVID-19 era due to heavy investments in digitalization and low margins that banks are facing (Carletti et al., 2020). Bank mergers can result in more efficient banks and a sounder banking system, which should lead to greater access to credit at lower cost and thus be beneficial for local communities Farrell & Shapiro (1990).^1^ However, the benefits of M &As can be offset if M &As make local banking markets less competitive and reduce the communities’ access to banking services and credit. Although banking regulatory agencies monitor M &As and do not approve those that are expected to result in uncompetitive banking markets, they stress out that more research is needed on the *ex*-*ante* optimal assessment of the net effect of bank mergers on both consumer welfare and soundness of the banking system (Vives, 2016).

This study presents a novel econometric method to evaluate and compare *ex*-*ante* the risk adjusted efficiency gains or losses in real money terms of a potential M &A activity that can be applied to any micro-study and to any industry. In this way, we contribute to the literature by creating a tool for policymakers to evaluate and approve or encourage industry consolidation.^2^ This is of imperative importance for two main reasons: First, due to the numerous cases of bank M &A that we witnessed worldwide after the onset of the global financial turmoil, and the accelerating competition from shadow banks and new digital entrants that has challenged the traditional business model in banking and could trigger a new wave of consolidation activity. Second, because the efficiency of the banking system is one of the major issues preoccupying the financial establishment as it is at the heart of a country’s financial system. It is generally accepted that efficient bank operations, which are linked to financial stability, allow entrepreneurs and households to enjoy higher-quality services at lower costs (European Commission 2014). Thus, measuring the efficiency of banking institutions and analyzing the factors that explain it is very important for supervisory authorities in order to design the regulatory framework and for bank management to draw up their business plans. It is indeed necessary to identify the nature of inefficiencies. These can occur due to information on the most effective processes not being easily accessible, free, or accurate. This has a direct impact on the time needed for each credit institution to respond to changes in environmental or market conditions. Therefore, the effect that inefficiencies have on organizational learning is significant and constitutes an important source of differences among financial institutions as they can create a competitive advantage in the long run.

Nevertheless, surveys on bank efficiency implicitly draw conclusions based on the assumption that all banks in a sample use the same production technology. Neglecting the existence of unobserved differences in technological regimes can have distorting effects on efficiency estimates by incorrectly assigning these deviations to inefficiency (Koetter and Poghosyan, 2009). In this study, we approach this consideration by estimating the unobserved heterogeneity in banking technologies using a Latent Class Stochastic Frontier Model (LCSFM). In this way, we manage to identify different technological regimes within a country’s banking system, and more importantly, we reveal the classification of each financial institution into these regimes. This triggers the aim of our paper, which is to measure efficiency gains or losses in real money terms of a prospective (i.e., before it is realised) bank consolidation activity that consists of financial institutions that may belong to either different or the same technological regime.^3^ We argue, that the *ex*-*ante* empirical assessment of efficiency of a potential M &A in real money terms is of vital importance for the economy, as it can provide policymakers with additional insights regarding the realised socio-economic benefits that a consolidation activity may have.^4^

Moreover, we contribute to the strand of the literature regarding the role of strategic vision and fit for a successful postmerger integration (Cartwright and Cooper, 1993, 1996; Yetton and Johnston, 1996; Epstein, 2005; Ishii and Xuan, 2014; Schmidt, 2015; Renneboog and Vansteenkiste, 2019). Corporate strategists have long recognised that the strategic fit between merging partners is a critical factor in determining the success or failure of a deal (Levine and Aaronovitch 1981; Lubatkin 1983; Markides 1992). To this end, we employ the similarity index of Altunbas and Marques-Ibanez (2008)^5^ who analysed the impact of M &As on performance in the European Union banking sector according to the similarities between target and bidder. If two firms show similar resource allocation patterns, measured from their balance sheet statements, across a variety of strategically relevant characteristics, they can be broadly considered to be strategically similar (Harrison et al., 1991). Thus, we are able to examine the strategic similarities/differences between potential banks’ M &As that lead to cost risk adjusted efficiency gains/losses.

In order to amplify the validity of our inferences, we examine two very different banking systems in terms of their level of sophistication. First, we focus our attention on the UK banking system, which is very complex with an advanced capital market. Its financial institutions have expanded their roles beyond their traditional payment services, intermediation between savers and borrowers, and insurance against risk function by adopting a more universal type of banking. The members of the UK banking system are of major importance to public authorities, as they were among the first credit institutions to suffer the impact of the global financial meltdown. The consequences of the crisis were severe not only for the UK’s public finances and capital market but also for the financial segments and public sectors of places that UK financial institutions are interconnected with.^6^ The second country of interest is Greece, where the stability of its simpler banking sector and its role as a financial intermediary has been distorted by the the second wave of the global economic crisis, the Sovereign Debt Crisis. As in the case of the UK, fiscal authorities intervened and tried to recapitalize Greek banks.^7^ However, that was not enough for the Greek banks to withstand the augmented and more frequent cracks from the debt crisis, as they were the main holders of the so called “toxic” government bonds^8^ whose value decreases every day. In turn, the more the increase in the country’s public debt, the more fragile the nation’s banks become.^9^

The fundamental differences in the structure and the impact that the global financial turmoil had on the two disparate banking systems, motivate us to conduct an empirical analysis in order to identify efficiency benefits, resulting from prospective M &As, which can reduce the scale of state intervention (i.e., bailouts) and in turn alleviate the taxpayers’ burden. Therefore, we are able to deduce some common policy implications for both the UK and Greece in line with the post-Brexit era and the on-going creation of a unique European banking regulatory framework, the so-called Capital Requirements Directives (CRD IV)^10^ package of the European Banking Authority (EBA).

We present empirical evidence of enhanced efficiency and cost reductions in real money terms that could lead to tax benefits as a result of potential consolidation activities. Most of the potential banks’ M &A that generate cost efficiency surplus are among institutions with similar capitalisation, loan, deposit and earnings strategies. Our empirical findings show that bank heterogeneity in both banking markets can be captured when a model with two classes is estimated. We find that in both countries, the financial institutions that belong to the first technological regime, are well capitalized, possess superior management of both credit and liquidity risk and are proved to be the most efficient. Furthermore, regarding the banking institutions that belong to the second class of both countries, we argue that potentially higher efficiency levels could be achieved as a result of future M &A activity among them. Finally, regarding the Greek banking sector specifically, we present evidence of decreased efficiency in two of the four new “cornerstones” of the Greek economy, to which the country’s economic recovery has been attributed, due to their particular consolidation decisions.

The rest of the paper is organized as follows. Section 2 provides an overview of the theoretical framework and presents the empirical model. Section 3 describes the data and specifies the model. Section 4 presents and discusses the empirical evidence of applying the models to the UK and Greek banking sectors and includes the findings regarding the proposed methodology of recent and potential M &A activity in both banking systems. Conclusions and insights for future research are presented in the final section.

## Stochastic frontier analysis

Investigating the efficiency measurement literature, it is evident that stochastic production (or economic) frontier functions have been increasingly used to measure the efficiency of individual producers. Notably, they seem to dominate parametric approaches (Kumbhakar and Lovell, 2000). In particular, the Stochastic Frontier Approach (SFA) separates inefficiencies from random noise; however, it needs an a priori assumption on the error term as a prerequisite. The alternative parametric techniques, such as the Distribution Free Approach (DFA) (Berger, 1993) and the Thick Frontier Approach (TFA) (Berger and Humphrey, 1991), may require less structure on the error term, but they impose an assumption of constant core inefficiency or do not present bank-specific point estimates. On the contrary, non-parametric techniques, while they do not impose any assumption on the error term, do not take into consideration the random noise and have an extreme sensitivity to outliers. In the present study, we follow several empirical works in the literature and use SFA to estimate the efficiency of banks (Kumbhakar, 1990, 1997; Resti, 1997; Fiordelisi et al., 2011).

The stochastic frontier production function was independently proposed by Aigner et al. (1977), Battese and Corra (1977), and Meeusen and Broeck (1977) and was applied to banking by Ferrier and Lovell (1990). It takes the following general form:

1 $$\begin{aligned} y=\beta ^{^{\prime }}x+v-u \end{aligned}$$

where *y* is the observed outcome (goal attainment), $\beta ^{^{\prime }}x+v$ is the optimal stochastic frontier goal followed by the individual, $\beta ^{^{\prime }}x$ is the deterministic part of the frontier, and $v\sim N[0,\sigma _{v}^{2}]$ is the stochastic part. A stochastic frontier is created if we combine these two parts. The aggregate amount of deviation from the optimum that lies on the frontier is what constitutes *u*.

Economic representations of production technology include cost, revenue, and profit frontiers. These economic frontiers are then used as standards against which to measure cost, revenue, and profit efficiency. As described by Kumbhakar and Lovell (2000), a cost stochastic frontier takes the form:

2 $$\begin{aligned} c\left( y_{i},w_{i};\beta \right) \end{aligned}$$

and can be written as

3 $$\begin{aligned} C_{i}\ge c\left( y_{i},w_{i};\beta \right) \cdot \exp \left\{ v_{i}\right\} , \end{aligned}$$

where $$c\left( y_{i},w_{i};\beta \right) \cdot \exp \left\{ v_{i}\right\} $$ is the stochastic frontier and $$C_{i}$$ is the observed cost. The stochastic cost frontier consists of two parts: the $$c\left( y_{i},w_{i};\beta \right) $$ part, which is the deterministic kernel and is the same for all producers, and the $$\exp \left\{ v_{i}\right\} $$ part, which is unique to each producer and captures the effects of random shocks on each producer. To be more specific, $$\beta $$ is a vector of technology parameters to be estimated, $$ y_{i}$$ and $$w_{i}$$ indicate vectors of output and input prices, respectively, and $$v_{i}$$ is a producer-specific random disturbance. The measure of cost efficiency is then

4 $$\begin{aligned} CE_{i}=\frac{c\left( y_{i},w_{i};\beta \right) \cdot \exp \left\{ v_{i}\right\} }{C_{i}}\text {.} \end{aligned}$$

This is the ratio of the minimum possible cost, given $v_{i}$, to actual total cost. If $C_{i}=c\left( y_{i},w_{i};\beta \right) \cdot \exp \left\{ v_{i}\right\}$, then the firm *i* is fully efficient and $CE_{i}=1$ . Otherwise actual cost exceeds the minimum so $0\le CE_{i}\le 1$.

A number of different functional forms are used in the literature to model production functions such as *Cobb–Douglas* which is log linear in outputs and inputs, the *Translog *function which is a generalization of a *Cobb–Douglas* function, a* Quadratic* in inputs function and a *Normalised* quadratic function. The first two are the most widely used in the literature. Assuming that the stochastic cost frontier follows a *Cobb–Douglas *function its log form representation can be written as

5 $$\begin{aligned} \ln \hbox {Ci}\ge & {} \ln c\left( y,w_{i}\right) +v_{i} \nonumber \\= & {} \ln c\left( y,w_{i}\right) +u_{i}+v_{i} \end{aligned}$$

where $\left( u_{i}\right)$ is a nonnegative inefficiency component. Cost efficiency is then $CE_{i}=\exp \left\{ -u_{i}\right\}$. Aigner et al. (1977) assume $v_{i}\sim N\left[ 0,\sigma _{\nu }^{2}\right]$ and $u_{i}\sim N\left[ 0^{+},\sigma _{u}^{2}\right]$. In addition to the half-normal assumption for $u_{i}$, other one sided-distributions have been used including the truncated -normal, where $u_{i}\sim$ *iid* $N\left[ \mu ,\sigma _{u}^{2}\right]$ introduced by Stevenson (1980), the exponential where $u_{i}\sim$ *iid* $\exp onetial$ introduced by Aigner et al. (1977) as well as Meeusen and Broeck (1977), and gamma where $u_{i}\sim$ *iid* *gamma* introduced by Greene (1980a, 1980b) and Stevenson (1980).

## Technological heterogeneity

The estimation of a stochastic frontier function imposes a strong assumption that the underlying production technology is common to all producers. Neglecting the existence of different technologies in banking can contaminate efficiency, market power, and other performance measures. An important drawback of the homogeneous technological regime assumption is that it imposes restrictions on certain important characteristics of banking technology, such as technical progress and scale economies. That is, the estimate of the underlying technology may be biased. Thus, unobserved technological differences are not taken into account during the estimation procedure, and consequently, the effects of these omitted unobserved technological differences might be inappropriately labelled as inefficiency.

Despite the on-going harmonization of regulation, very different banks continue to exist side by side. In the literature on bank efficiency, we can identify two types of systematic differences across and within national banking markets. The first type of heterogeneity refers to the environment in which banks operate, which is exogenous to managers. Conditional on environmental differences, banks may employ different business models (retail versus wholesales) that require different intermediation technologies. The second type of systematic differences refers to managerial choices, especially those related to risk management, which affect the banking firm’s efficiency (Kauko, 2009). This second type of heterogeneity is identified as endogenous to managers and influences the ability to attain the optimum benchmark rather than the shape of the efficient frontier.

### Methods to account for heterogeneous production technologies

There are several approaches that can be employed to capture technological differences. One approach is the one introduced by Hayami and Ruttan (1970) which, based on the notion of the metafrontier, emanates from the metaproduction function. This approach still remains an extremely ambiguous notion, due to the fact that it is not conducive to the understanding of the marginal contribution of the different elements of environmental factors that might shed light on the differences in bank efficiency. Another approach is to include country-specific environmental variables that are likely to influence technologies of banks, such as the level of economic development and institutional background, as additional explanatory variables in the frontier (Bonin et al., 2005; Berger, 2007). The main disadvantage of this approach is that the introduction of the environmental variables only affects the intercept of the frontier specification, leaving the slope unaffected (Bos and Schmiedel, 2007). Another drawback of this approach is that technological differences are assumed to be country-specific, which rules out the possibility that banks located within the same country may employ different business models (Koetter and Poghosyan, 2009). An alternative approach that attempts to relieve the impact of technological differences is a priori sample separation. The sample separation can be based, for instance on the organizational structure of banks Mester (1993); Altunbas et al. (2001), or their geographical location (Mester, 1996; Bos and Schmiedel, 2007; Claessens et al., 2001). In this approach the main disadvantage is that a priori restriction of sample separation is to some extent arbitrary. For instance, Koetter and Poghosyan (2009) show that even banks having similar organizational structure can operate under different technological regimes.

### Latent class stochastic frontier model

In this study, we account for differences in technological regimes using a latent class stochastic frontier model (LCSFM), which addresses the disadvantages associated with the aforementioned alternative approaches. Unlike the first of these approaches, the impact of the environmental factors is not only reflected in the magnitude of the intercepts, but also affects the slope coefficients. Thus, we can have two different impacts on the stochastic frontier. First we may have parallel shifts of the frontier and second we may have systematic different deviations from the frontier. Specifically, the environmental variables enter as latent class determinants rather than as a part of the frontier and thus influence both estimates of the technological regime of banks and their cost efficiency simultaneously. Unlike the second approach described earlier, the latent class method does not require a priori grouping of banks. Instead, it utilizes all information available in the sample and identifies separate technological regimes based on the maximum likelihood principle.

There are some notable contributions in the literature that combine mixed latent class principles with the SFA. One strand of the literature consists of a Bayesian approach in allocating firms to different technological regimes. To be more precise Tsionas and Kumbhakar (2004) propose a stochastic frontier production function augmented with a Markov switching structure to account for different technology parameters across heterogeneous countries. Another strand lies in the principles of Maximum Likelihood approach. Specifically, Greene (2002) proposes a maximum likelihood LCSFM using sample separation information and allowing for more than two classes. Another noteworthy study as well the study is Gaudill (2003)^11^ who proposes an expectation-maximization (EM) algorithm and without having sample separation information, he estimates a combination of two stochastic cost frontiers (see Greene 2001). Both of the previous studies do not allow to the efficiency term to vary every year, which is an important drawback when we conduct productivity growth studies. This obstacle is surmounted in our analysis, as we use panel data LCSFM for the estimation of our latent class efficiency determinants. This is an approach employed in banking studies by Orea and Kumbhakar (2004) and Poghosyan and Kumbhakar (2010). However, these studies assume that every bank in the sample remains in the same technological regime for all the years it operates (Bos et al., 2010). The novelty of our study is that it uses two methodologies proposed in the literature. First, we apply the one used by Orea and Kumbhakar (2004) that allows for a time-varying efficiency term. Second, as a robustness check of our estimates, we apply the methodology followed by Bos et al. (2010), which permits the financial institution to be in one regime in a specific year and in another regime the year after. Thus, the first methodology adopts a panel-based approach, whereas the second one treats the data set as a pooled cross-section. To the best of our knowledge, this is the first time in the latent class stochastic frontier literature that both models would be applied to answer the same research question. Thus, we manage to surmount several modelling limitations and are able to produce the most accurate comparisons and inferences.

In determining efficiency, the technology of banks belonging to each class must be modelled. Following Orea and Kumbhakar (2004), we assume that the technology is represented by a cost function. This may be written for class *k* as

6 $$\begin{aligned} \ln C_{it}=\ln C(y_{it},w_{it},t\text { };\beta _{k})+u_{it\mid k}+v_{it\mid k}, \end{aligned}$$

where subscripts $$i=1,....N$$, $$t=1,....,T_{i}$$ and $$k=1,...,K,$$ stand for bank, time and class respectively. $$C_{it}$$ is individual bank total cost; $$ y_{it}$$ and $$w_{it}$$ indicate vectors of output and input prices; and $$ \beta _{k}$$ is a class-specific vector of parameters to be estimated. The two-sided random error term $$v_{it\mid k}$$ is assumed to be independent of the non-negative cost efficiency variable $$u_{it\mid k}$$ for each class. Here the technology is represented by a dual cost function.

To estimate the model using maximum likelihood we employ standard distributional assumptions (Orea and Kumbhakar, 2004; Poghosyan and Kumbhakar, 2010), where the random error term is assumed to be *i*.*i*.*d* for each class *k* and follows a normal distribution with zero mean and constant variance $\sigma _{vk.}^{2}$.The inefficiency term $u_{it\mid k}$ is modeled as the product of a time-invariant firm effect $u_{i\mid k}$ and a non-negative deterministic parametric function of time $\psi _{it}$ and other explanatory variables $z_{it}$. The term $u_{i\mid k}$ is assumed to follow a truncated normal distribution with zero mean and constant variance $\sigma _{uk\text {.} }^{2}$. In line with Orea and Kumbhakar (2004) and Poghosyan and Kumbhakar (2010) we specify cost inefficiency $u_{it\mid k}$ as:

7 $$\begin{aligned} u_{it\mid k}=\psi _{it}(z_{it}^{^{\prime }}\eta _{k})\cdot u_{i\mid k} =e^{(z_{it}^{^{\prime }}\eta _{k})}\cdot u_{i\mid k} \end{aligned}$$

where, $u_{i\mid k}\ge 0$; $\eta _{k}=(\eta _{1k,...,} \eta _{Hk})^{^{\prime }}$ is a *H* *x* 1 vector of parameters and $z_{it}=(z_{1it,...,}z_{Hit})^{^{\prime }}$ is a *H* *x* 1 vector of determinants of cost inefficiency.

The likelihood function $\left( LF\right)$ for firm *i* belonging to class *k* for all time periods^12^ (see Orea and Kumbhakar 2004; Poghosyan and Kumbhakar 2010) is:

8 $$\begin{aligned} LF_{ik}(\theta _{k})= & {} \ln [1-\phi (-z_{i}^{*})]+(z_{i}^{*})^{2} -\frac{1}{2}[\ln 2\pi +\ln \sigma _{k}^{2}]\cdot T_{i}-\frac{1}{2} \ln (1-\lambda _{k})\cdot (T_{i}-1) \nonumber \\{} & {} -\frac{1}{2}\cdot \ln \left[ 1+\lambda _{k}\cdot \left( \sum _{t=1}^{T_{i}} \psi _{it}(\eta _{k})^{2}-1\right) \right] -\frac{1}{2}\cdot \sum _{t=1}^{T_{i}}[\varepsilon _{it}(\beta _{k})^{2}/(1-\lambda _{k})\sigma _{k}^{2}] \end{aligned}$$

where

$$\begin{aligned} z_{i}^{*}=\frac{\lambda _{k}\cdot \sum _{t=1}^{T_{i}}\psi _{it} (\eta _{k})\cdot \varepsilon _{it}(\beta _{k})}{\left\{ \lambda _{k}\cdot (1-\lambda _{k})\cdot \sigma _{k}^{2}\cdot \left[ 1+\lambda _{k}\cdot \left( \sum _{t=1}^{T_{I}}\psi _{it}(\eta _{k})^{2}-1\right) \right] \right\} ^{1/2}}, \end{aligned}$$

$\varepsilon _{it}=\varepsilon _{it}(\beta _{k})=\ln C_{it}-\ln C(y_{it},w_{it},t$ $;\beta _{k})$; $\sigma _{k}=\left[ \sigma _{vk}^{2}+\sigma _{uk}^{2}\right] ^{\frac{1}{2}};$ $\lambda _{k}=\sigma _{uk}/\sigma _{\nu k}$, the $\lambda _{k}$ parameter is the ratio of the standard deviation of the one-sided inefficient component to the standard deviation of the two sided random error; and $\theta _{k}=\left( \beta _{k},\sigma _{k}^{2},\lambda _{k},\eta _{k}\right)$ are the parameters associated with the technology of class *k*, and $\phi (\cdot )$ denotes the standard normal distribution function.

The unconditional likelihood of bank *i* is obtained as a weighted sum of the *k*-class likelihood functions, where the weights are the class membership probabilities reflecting the uncertainty regarding the true membership in the sample:

9 $$\begin{aligned} LF_{i}\left( \theta ,\delta \right) =\sum _{k=1}^{K}LF_{ik} \left( \theta _{k}\right) \cdot P_{ik}\left( \delta _{k}\right) \end{aligned}$$

where $$0\le P_{ik}\le 1$$ and $$\sum _{k=1}^{K}P_{ik}=1$$

We can parameterize the class probabilities by employing the multinomial logit model:

10 $$\begin{aligned} P_{ik}\left( \delta _{k}\right) =\frac{e^{\left( \delta _{k}^{\shortmid } q_{i}\right) }}{\sum \nolimits _{k=1}^{K}e^{\left( \delta _{k}^{\shortmid }q_{i}\right) }} \end{aligned}$$

where $k=1,...,K$, denotes classes; $\delta _{1}=0$ is a parameter normalization for the reference class and $q_{i}$ is a vector of bank-specific and time-invariant class determinants.

Combining Eqs. (8)–(10), the overall likelihood function is a continuous function of the vectors of parameters $\theta$ and $\delta$ and is indicated as:

11 $$\begin{aligned} \ln LF\left( \theta ,\delta \right) =\sum \limits _{i=1}^{N}\ln LF_{i} \left( \theta ,\delta \right) =\sum _{i=1}^{N} \ln \left\{ \sum _{k=1}^{K}LF_{ik}\left( \theta _{k}\right) \cdot P_{ik}\left( \delta _{k}\right) \right\} \end{aligned}$$

The estimated parameters can then be used to compute the conditional posterior class probabilities. Greene (2002) showed that the posterior probability of class-*k* membership for bank *i* can be computed as:

12 $$\begin{aligned} P\left( k\mid i\right) =\frac{LF_{ik}\left( \theta _{k}\right) \cdot P_{ik}\left( \delta _{k}\right) }{\sum \nolimits _{k=1}^{K}LF_{ik} \left( \theta _{k}\right) \cdot P_{ik}\left( \delta _{k}\right) } \end{aligned}$$

Unlike the standard SFA, where the cost frontier is the same for each bank, in the LCSFM, we estimate several frontiers (equal to the number of classes).

What remains to be estimated is the cost inefficiency term in the case when we have several benchmarks. According to Greene (2002), we can achieve that by getting the weighted average of the cost inefficiency terms:

13 $$\begin{aligned} \ln EF_{i}=\sum \limits _{k=1}^{K}P\left( k\shortmid i\right) \cdot \ln EF_{i}\left( k\right) , \end{aligned}$$

where $EF_{i}\left( k\right)$ is the bank’s cost efficiency using class-*k* technology as a reference. In this case technologies from every class are taken into account when estimating the cost efficiency.

## Data

### UK & Greek banking market

We now turn to our data characteristics. For the estimation of the model, we use data that consist of an unbalanced panel of all the financial institutions that provided credit during the years 1988–2011 in the UK and 1993–2011 in Greece.^13^ Overall, both our samples account for a significant market share in terms of assets, loans, and deposits, occasionally even more than 90% in each respective category in both countries.^14^ The number of banks we examine in our study changes during the sample period in both countries. This occurs specifically in Greece due to the many M &A that took place at the end of the 1990s (Rezitis, 2010; Tziogkidis et al., 2018). The observed wave of M &A events was triggered primarily by the willingness of the small banks to obtain a higher market share and secondarily by the privatization process initiated by the government, in line with the second Banking Directive. Table 1a and b provide an overview of some important banking indicators of the UK and Greek banking sectors for the whole period of our study.

**Table 1:** (a) UK—Time Series Analysis of characteristic banking indicators, (b) Greece—Time Series Analysis of characteristic banking indicators

Year	Num OBS	T.A (B)	Gr. Ls (B)	Dep. (B)	Eqt. (B)	L.L.P (M)	HHI
(a)							
1988	13	10.73	18.09	9.58	0.55	25.77	0.19
1989	40	16.6	26.98	14.33	0.86	338.95	0.12
1990	49	19.4	36.34	16.76	0.96	205.41	0.08
1991	53	21.63	37.9	18.64	1.11	287.72	0.08
1992	66	17.16	25.94	14.39	0.87	227.15	0.08
1993	69	15.95	23.62	13.04	0.81	147.54	0.07
1994	70	19.92	31.13	15.9	1.01	76.32	0.08
1995	80	14.56	22.57	11.56	0.89	45.88	0.06
1996	110	14.76	25.06	11.75	0.92	30.11	0.05
1997	114	18.04	29.84	14.22	0.99	38.9	0.08
1998	115	20.52	34.13	16.16	1.16	100.34	0.06
1999	116	18.3	29.59	14.47	1.2	73.44	0.05
2000	117	24.06	35.9	18.94	1.7	67.05	0.07
2001	120	23.65	34.3	18.73	1.77	95.16	0.06
2002	125	33.11	53.37	26.58	2.05	127.42	0.07
2003	127	35.3	63.01	27.02	2.76	137.02	0.06
2004	127	73.56	142.07	59.93	5.16	351.77	0.15
2005	126	87.6	150.83	62.92	4.42	223.69	0.12
2006	121	104.11	204.36	68.12	6.32	541.66	0.14
2007	120	132.24	264.95	98.8	8.22	579.07	0.23
2008	116	107.92	157.32	53.52	4.1	783.78	0.09
2009	116	87.82	142.22	53.25	7.16	971.87	0.08
2010	113	86.56	135.5	51.52	7.32	675.16	0.07
2011	101	138.39	213.96	80.69	10.43	863.94	0.08
Total	2324	1141.89	1938.98	790.82	72.74	7015.12	0.09
(b)							
1993	19	3.84	5.24	3.28	0.17	12.91	0.21
1994	19	4.85	6.89	4.18	0.22	18.54	0.23
1995	19	6.05	8.7	5.25	0.26	13.78	0.21
1996	21	5.04	6.95	4.49	0.24	24.62	0.16
1997	21	5.74	6.92	5.07	0.27	32.97	0.2
1998	20	6.79	8.19	6.06	0.42	41.5	0.16
1999	16	8.77	9.1	7.47	0.9	45.36	0.16
2000	15	9.31	8.77	8.04	0.83	38.31	0.16
2001	15	9.94	8.76	8.77	0.76	44.99	0.17
2002	18	9.85	10.33	8.76	0.6	47.85	0.18
2003	20	11.84	14.96	10.17	0.81	75.79	0.16
2004	21	13.33	18.15	10.83	0.79	89.34	0.15
2005	21	13.44	15.86	10.93	0.93	75.35	0.14
2006	19	19.2	25.29	15.08	1.39	125.15	0.14
2007	19	26.95	39.68	19.55	2.27	120.8	0.13
2008	19	31.71	44.12	25.05	2.13	260.27	0.14
2009	19	34.67	49.95	28.1	2.85	424.91	0.14
2010	20	30.36	40.57	24.77	2.74	562.62	0.13
2011	15	30.54	39.51	26.21	1.1	1779.96	0.19
Total	356	282.22	367.94	232.06	19.68	3835.02	0.17

(a) Presents an overview of the UK banking system throughout our sample period. T.A, Gr. Ls, Dep., Eqt, L.L.P, HHI represent average values of Total Assets, Gross loans, Deposits, Equity, Loans and loss Provisions and Market Concentration (expressed by the Herfindahl-Hirschman (HHI) Index and it is defined as the sum of the squares of the market shares of all banks in the sample for each year: a HHI index below 0.01 indicates a highly competitive index, a HHI index below 0.15 indicates an unconcentrated index, a HHI index between 0.15 and 0.25 indicates moderate concentration, while a HHI index above 0.25 indicates high concentration.) respectively. ‘B’ stands for billions while ‘M’ for millions

(b) Presents an overview of the Greek bank ing system throughout our sample period. T.A, Gr. Ls, Dep., Eqt, L.L.P, HHI represent average values of Total Assets, Gross loans, Deposits, Equity, Loans and loss Provisions and Market Concentration (expressed by the Herfindahl-Hirschman (HHI) Index and it is defined as the sum of the squares of the mark et shares of all bank s in the sample for each year: a HHI index below 0.01 indicates a highly competitive index, a HHI index below 0.15 indicates an unconcentrated index, a HHI index between 0.15 and 0.25 indicates moderate concentration, while a HHI index above 0.25 indicates high concentration.) respectively. ‘B’ stands for billions while ‘M’ for millions

### Model specification

The LCSFM (Orea and Kumbhakar, 2004) presented in the previous section requires the following three sets of variables to be determined:

#### Main variables

A critical discussion of the two most widespread approaches for measuring and defining inputs and outputs is that by Berger and Humphrey (1997). They conclude that despite the fact that none of the approaches is ideal, the production approach is preferable when we want to evaluate the efficiency of financial institutions’ branches, whereas the intermediation approach is preferable when we want to analyze the efficiency of the whole financial institution. With this in mind, to define outputs and input prices we follow the intermediation approach (Sealey and Lindley 1977; Hughes and Mester, 1998, 2011; Koetter et al. 2012; Delis et al., 2014; Degl’Innocenti et al. 2017; Tsionas and Philippas 2021). Under this approach, a bank uses labor and physical capital to attract deposits, which in turn are used to fund loans and other earning assets.^15^ Therefore, we specify the two mainstream types of outputs as total loans $\left( y_{1}\right)$ and other earning assets $\left( y_{2}\right)$ and the three mainstream types of input prices as the ratio of interest expenses to total deposits $(w_{1})$, the ratio of staff expenses to the number of employees $(w_{2})$ and the expenses on fixed assets to total fixed assets $(w_{3})$. We also account for total off-balance sheet items (OBS), as an additional output $\left( y_{3}\right)$.^16^ Furthermore, following Berger and Mester (1997), we include equity capital (*EC*) to control for differences in risk preferences, which may arise due to regulation, financial distress, or informational asymmetries.^17^ We also account for the risk exposure of the bank by considering the nonperforming loans to total loans (*NPLs*). Finally, we include a time trend $\left( T\right)$ to capture the potential technical change that occurred during the examination period for each financial institution, while as dependent variable we use total cost $\left( TC\right)$.^18^

Following the majority of empirical studies in banking (e.g. Fiordelisi et al. 2011; Philippas et al. 2015; Casu et al. 2016; Baltas et al. 2017; Clark et al. 2018; Tziogkidis et al. 2018), we obtain our bank-level data from the Bankscope database of the Bureau Van Dijk company. We also obtain detailed information on M &A from the Zephyr database of the Bureau Van Dijk company.^19^ All data are deflated using each country’s GDP deflator (with 2011 as the base year) obtained from the World Bank database and converted to US dollars. We exclude observations for which data on any of the variables used in our study is missing. (Lozano-Vivas and Pasiouras, 2010). Moreover, following Berger and Mester (1997) and Delis et al. (2014), we apply an outlier rule to the variables used, corresponding to the 5th and 95th percentiles of the distributions of the respective variables.^20^ Our final samples account for 124 financial institutions and 1,856 observations for the UK banking sector and for 30 financial institutions and 356 observations for the Greek banking sector.

#### Inefficiency determinants

Turning our attention to the parametric part of the inefficiency component, we consider three $(z_{it})$ variables, for each banking sector.

**Time** The first variable is time, indicating spillover effects from developments, such as deregulation processes and the transfer of know-how. The parametric component becomes a function of time with only one parameter. In turn, efficiency either increases, decreases, or remains constant. We use the time trend to measure time.

**Size** The second variable is *size*, reflecting debates concerning the optimum size a financial institution should be. In general, this variable is supposed to have a positive effect on efficiency as it increases to a certain level. Nevertheless, the impact of an extremely large size can be proved to be counterproductive for the credit institution’s efficient operation. According to empirical findings, the relationship between efficiency and size is not linear. We use each bank’s real assets to measure this determinant.

**Type and Ownership** The third variable is different for each country. In the UK, we recognize that two different types of financial institutions dominate the provision of credit: banks and building societies. Therefore, we create a dummy variable, *bs*, which takes the value of 1 if the financial institution is a building society and 0 otherwise. Regarding the Greek banking sector, a key development we take into account is the increase in the number of privately owned institutions. We examine the impact of privately and publicly owned or government-owned banks on bank efficiency. The efficiency of the banking industry can benefit from the fact that privately owned banks perform more efficiently compared to their rivals, who often operate on different business plans due to the meddling of politicians in the banks’ affairs (see La Porta et al. 2002). There is empirical evidence supporting this hypothesis, particularly for the period in which the share of the publicly owned banks is very high and their performance is critical for the Greek financial system (Delis and Papanikolaou, 2009). We control for the effects using a dummy variable *owner* that takes the value of 1 if the depository institution is privately owned and 0 otherwise.

#### Class membership determinants

We consider the firm-average value of five variables, apart from an intercept, as determinants of the latent class probabilities. As customary in cluster analysis, the variables included in the class probabilities are five balance sheet ratios.

**Capital adequacy** Examining the annual reports of the governors of both countries’ central banks, we notice that the financial institutions are quite heterogeneous in terms of capital requirements. According to the literature, credit institutions that have a significant amount of capital are considered more stable, can implement high-cost plans to ameliorate their economies of scope, and are able to achieve this in a safer way by reducing the potential risks. Furthermore, they can adjust better to unexpected developments. In addition, shareholders of banks that are well capitalized can reduce moral hazard by controlling the bank’s management more closely. We expect the most efficient banks to have higher levels of capital. In order to measure the capital adequacy, we use the equity to assets ratio.

**Liquidity risk** The last financial turmoil demonstrates the severe impact that this risk can have on the financial system. Clearly, credit institutions with high liquidity are able to expand and/or face potential adverse conditions in the economic environment better than those that need to resort to stock markets to raise funds, especially at times of worsening conditions in money markets like the one we experienced in the GFC. Although liquidity risk can be measured in different ways, we follow the approach by Altunbas et al. (2000) and measure it using the loans to assets ratio ratio. The higher this ratio, the greater the need of the financial institutions to raise finance.

**Credit risk** This specific determinant reflects a very important risk that depository institutions confront. An indication of the quality of the credit risk management of an institution stems from the level of this risk, given that high values are associated with less efficient lending procedures (Berger and DeYoung, 1997). That said, credit institutions seeking higher rents undertake risky projects in the expectation of higher yields. It can also be that borrowers face difficulties meeting their obligations due to unexpected adverse economic developments. Thus, high-value credit risk may not be attributable to poor management. Additionally, a financial institution may choose a strategy that reflects reduced efforts in granting and monitoring loans that may appear to be cost-efficient but that have an increased credit risk. We measure this specific category of risk by each bank’s provisions to total assets ratio.

**Service Concentration** We stress the different strategies that credit institutions follow to create their products. We carefully examine the income statements and identify substantial differences in the level of loans, securities, investment assets, and OBS activities. For this purpose, we measure each financial institution’s degree of specialization. We argue that there exists a trade-off between the variety of products and services that a bank offers and its efficiency level as in this case it requires a more specialized management. We measure it as the sum of the squared ratios of the value of each output to the total value of outputs of each financial institution.

**Profitability** All depository institutions’ annual income statements show tremendous differences regarding their profitability. This determinant can have opposite effects depending on which economic efficiency is the subject of interest. High profitability allows banks to invest in improved technology and in skilled personnel with higher wages as they expect this to result in much higher output gains and thus higher profit efficiency. However, higher wages and investments in advanced technology would mark an increase in costs, resulting in a decline in cost efficiency. We proxy the specific variable with the ratio of pre-tax profits to assets (ROA).^21^

Table 2a and b present descriptive statistics of the variables we use in the estimation of the cost frontier kernel, the inefficient component, and the regime class membership for the UK and Greek banking sectors.^22^ Even though we use natural logarithms of variables in the cost kernel components (these represent the intermediation technology) to compute the efficiency scores, we show the mean and standard deviations in levels to allow meaningful comparisons.

**Table 2:** (a) UK—Descriptive Statistics of the variables of interest, (b) Greece—Descriptive Statistics of the variables of interest

Variable		Mean	SD	Percentiles	
				5th	95th
(a)					
*Kernel determinants*					
Total cost	tc	1147.161	174.709	804.612	1489.709
Price of borrowed funds	w1	0.126	0.019	0.089	0.163
Price of labor	w2	0.023	0.001	0.021	0.025
Price of physical capital	w3	6.36	0.744	4.901	7.82
Total loans	y1	26,154.18	2781.631	20,700.58	31,607.78
Total earning assets	y2	21,727.69	2127.914	17,555.82	25,899.56
Off-balance sheet items	y3	14,404.49	1150.945	12,147.57	16,661.41
Equity capital	EC	2925.062	327.158	2283.656	3566.467
Non Performing Loans	NPLs	0.052	0.063	0.007	0.102
*Inefficiency determinants*					
Time	z1	14.375	0.092	14.194	14.556
Size	z2	48,946.8	4949.264	39,243.56	58,650.03
*Class determinants*					
Capital adeqaucy	q1	0.157	0.003	0.15	0.163
Liquidity risk	q2	0.511	0.005	0.502	0.521
Credit risk	q3	0.946	0.264	0.427	1.464
Service concentration	q4	0.566	0.004	0.559	0.573
Profitability	q5	0.024	0.013	0.021	0.089
(b)					
*Kernel determinants*					
Total cost	tc	392.932	38.422	317.365	468.499
Price of borrowed funds	w1	0.058	0.002	0.054	0.062
Price of labor	w2	0.017	0.0005	0.016	0.018
Price of physical capital	w3	1.549	0.303	0.952	2.146
Total loans	y1	6913.851	625.514	5683.612	8144.091
Total earning assets	y2	4248.469	369.007	3522.74	4974.198
Off-balance sheet items	y3	2899.264	384.447	2142.604	3655.925
Equity Capital	EC	812.078	73.574	667.383	956.773
Non Performing Loans	NPLs	0.117	0.072	0.009	0.216
*Inefficiency determinants*					
Time	z1	9.938	0.291	9.366	10.51
Size	z2	14,750.98	1378.103	12,040.71	17,461.25
*Class determinants*					
Capital adeqaucy	q1	0.1	0.005	0.09	0.11
Liquidity risk	q2	0.556	0.01	0.535	0.576
Credit risk	q3	0.127	0.036	0.056	0.197
Service concentration	q4	0.464	0.006	0.453	0.475
Profitability	q5	0.0016	0.0019	0.0022	0.0033

**Fig. 1:**
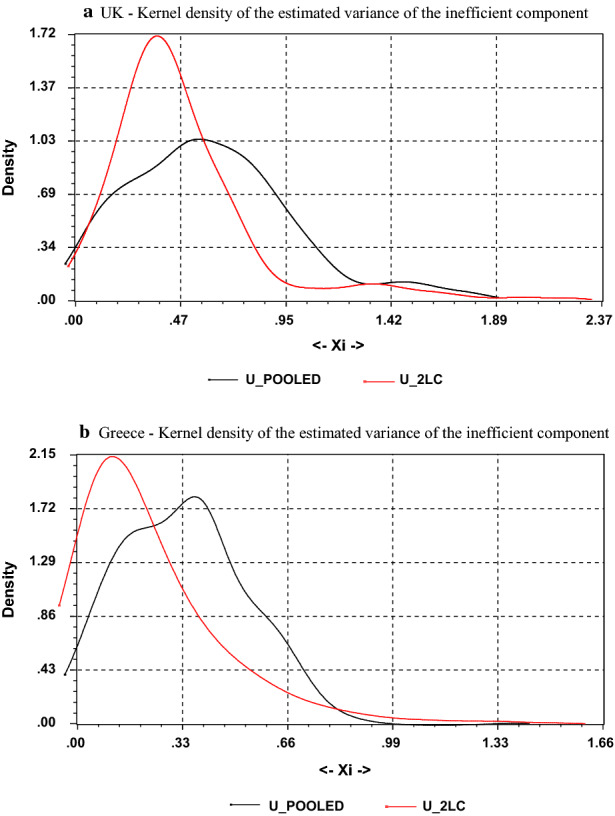
**a** UK—Kernel density of the estimated variance of the inefficient component. *Notes*: This figure displays the kernel density estimators for the two sets of the variance of inefficiencies $\{\sigma ^{2}u|k\}$ as far as the UK banking sector is concerned. The model is $lnC(it)=lnC(y[it],w[it],t ;\beta [k])+u[it|k]+v[it|k]$ where subscripts $i=1,....N$, $t=1,....,T\_\{i\}$ and $k=1,...,K$, stand for bank, time and class respectively. $C\{it\}$ is individual bank total cost; $y\{it\}$ and $w\{it\}$ indicate vectors of output and input prices; $\beta \{k\}$ is a class-specific vector of parameters to be estimated.The two-sided random error term *v*[*it*|*k*] is assumed to be independent of the non-negative cost efficiency variable *u*[*it*|*k*] for each class. Here the technology is represented by a dual cost function. $U\_POOLED$’ and ’$U\_2LC$’ refer to a model that assumes the same ($k=1$) production technology for all the banks in the sample and to a model with two ($k=2$) latent classes respectively. **b** Greece—Kernel density of the estimated variance of the inefficient component. *Notes*: This figure displays the kernel density estimators for the two sets of the variance of inefficiencies $\{\sigma ^{2}u|k\}$ as far as the Greek banking sector is concerned. The model is $lnC(it)=lnC(y[it],w[it],t ;\beta [k])+u[it|k]+v[it|k]$ where subscripts $i=1,....N$, $t=1,....,T\_\{i\}$ and $k=1,...,K$, stand for bank, time and class respectively. $C\{it\}$ is individual bank total cost; $y\{it\}$ and $w\{it\}$ indicate vectors of output and input prices; $\beta \{k\}$ is a class-specific vector of parameters to be estimated.The two-sided random error term *v*[*it*|*k*] is assumed to be independent of the non-negative cost efficiency variable *u*[*it*|*k*] for each class. Here the technology is represented by a dual cost function. $U\_POOLED$’ and ’$U\_2LC$’ refer to a model that assumes the same ($k=1$) production technology for all the banks in the sample and to a model with two ($k=2$) latent classes respectively

(a) Refers to 1856 observations and 124 UK financial institutions between 1988 and 2011. The table reports descriptive statistics of the kernel, inefficiency and the class membership variables we use in the estimation of the latent class stochastic cost frontier model (apart from the dummy variable that represents the type of the financial institution, i.e. ‘BS’) as described in Fig. 1a. All monetary variables are deflated using 2011 as a base year. Kernel determinants consist of the dependent variable, i.e. total cost (tc), inputs prices (w), output quantities (q), equity capital (EC) and non performing loans to total loans ratio (NPLs). Inefficiency determinants (z) consist of ‘Time’$=$ time-trend and ‘Size’ $=$ bank’s real assets. Finally the class ratio, determinants (q) consist of ‘Capital adequacy’ $=$ equity to assets ratio, ‘Liquidity risk’ $=$ loans to assets ratio, ‘Credit risk’$=$ loans loss provisions to total assets ratio and ‘Service Concentration’ $=$ the sum of the squared ratios of the value of each output to the total value of outputs of each financial institution

(b) Refers to 356 observations and 30 Greek financial institutions between 1993 and 2011. The table reports descriptive statistics of the kernel, inefficiency and the class membership variables we use in the estimation of the latent class stochastic cost frontier model (apart from the dummy variable that represents the onwership of the financial institution, i.e. ‘OWNER’) as described in Fig. 1b. All monetary variables are deflated using 2011 as a base year. Kernel determinants consist of the dependent variable, i.e. total cost (tc), inputs prices (w), output quantities (q), equity (EQ) and non performing loans to total loans ratio (NPLs). Inefficiency determinants (z) consist of ‘Time’ $=$ time-trend and ‘Size’ $=$ bank’s real assets. Finally the class determinants (q) consist of ‘Capital adequacy’ $=$ equity to assets ratio, ‘Liquidity risk’ $=$ loans to assets ratio, ‘Credit risk’ $=$ loans loss provisions to total assets ratio and ‘Service Concentration’ $=$ the sum of the squared ratios of the value of each output to the total value of outputs of each financial institution

The final specification of our latent class cost stochastic frontier model takes the following translog production function:^23^

14 $$\begin{aligned} \ln TC_{it}= & {} \beta _{0}+\sum \limits _{l=1}^{3}\beta _{yl}\ln y_{it,l}+\sum \limits _{s=1}^{2}\beta _{ws}\ln w_{it,s}+\frac{1}{2} \sum \limits _{l=1}^{3}\sum \limits _{s=1}^{2}\beta _{ylys}\ln y_{it,l}\ln y_{it,s} \nonumber \\{} & {} +\frac{1}{2}\sum \limits _{l=1}^{2}\sum \limits _{s=1}^{2}\beta _{wlws}\ln w_{it,l}\ln w_{it,s}+\sum \limits _{l=1}^{3}\sum \limits _{s=1}^{3}\beta _{ylws}\ln y_{it,l}\ln w_{it,s} \nonumber \\{} & {} +\left( \sum \limits _{s=1}^{2}\beta _{ws}\ln w_{it,s}\right) +\left( \sum \limits _{l=1}^{3}\beta _{ws}\ln y_{it,l}\right) +\beta _{EC}\ln EC_{it}+\beta _{NPLs}\ln NPLs_{it} \nonumber \\{} & {} +\beta _{t}T+\frac{1}{2}\beta _{tt}T^{2}+u_{it}+v_{it} \end{aligned}$$

where $k=1,...,K$, expresses class membership.

Inefficiency is modelled as a function of its determinants:

15 $$\begin{aligned} u_{it|k}=\exp ^{[\eta _{1i|k}TIME+\eta _{2i|k}SIZE+\eta _{3i|k}BS]} \end{aligned}$$

and

16 $$\begin{aligned} u_{it|k}=\exp ^{[\eta _{1i|k}TIME+\eta _{2i|k}SIZE+\eta _{3i|k}OWNER]} \end{aligned}$$

for the UK and Greek banking sectors, respectively.

*TIME*, *SIZE*, *BS*,  and *OWNER* refer to a *time-trend *variable, the *size* (in terms of assets) of each financial institution, a dummy variable reflecting the *type* of each UK financial institutions and the *ownership* of the Greek banks respectively.

The latent class probabilities are specified as:

17 $$\begin{aligned} P_{ik}\left( \delta _{k}\right) =\frac{e^{\left( \delta _{ok} +\delta _{1i|k}CAP\_ADEQ+\delta _{2i|k}LIQ\_RISK+\delta _{3i|k}CRED\_RISK +\delta _{4i|k}SERV\_CON+\delta _{5i|k}PROF\right) }}{\sum \nolimits _{k=1}^{K} e^{^{\left( \delta _{ok}+\delta _{1i|k}CAP\_ADEQ+\delta _{2i|k}LIQ\_RISK +\delta _{3i|k}CRED\_RISK+\delta _{4i|k}SERV\_CON+\delta _{5i|k}PROF\right) }}} \end{aligned}$$

where $CAP\_ADEQ,LIQ\_RISK,CRED\_RISK,SERV\_CON,and$ *PROF* refers to the *capital adequacy, liquidity risk, credit risk, service concentration* and *profitability* of each financial institution in both samples.

The estimated cost frontier must satisfy the following regularity conditions in order to ensure that is well behaved. There should be monotonicity and concavity in input prices. These two characteristics can only be checked after the estimation procedure of the model, whereas an additional one, linear homogeneity in input prices, has to be imposed a priori. The latter property requires:

18 $$\begin{aligned} \sum \limits _{s=1}^{3}\beta _{wsk}=1 \end{aligned}$$

Because the cost function is homogeneous of degree 1 in input prices, linear homogeneity restrictions are imposed on all price and cost variables with respect to one of the input prices. Here, we use the price of the physical capital depreciation and amortization $\left( w_{3}\right)$ as a numeraire. Lastly, we normalize the dependent variable and output quantities by equity capital.^24^

### Similarity index

Following Ramaswamy (1997) and Altunbas and Marques-Ibanez (2008), we measure the strategic similarity of firms involved in M &A activity by using a simple indicator containing the financial characteristics for each strategic variable and individual consolidation activity:

19 $$\begin{aligned} SI_{m,s}=\sqrt{(X_{A,m,s}-X_{B,m,s})^{2}} \end{aligned}$$

where $SI_{m,s}$ is the similarity index for the *s*th variable for the *m*th M &A, and $X_{A,m,s}$ and $X_{B,m,s}$ are the scores of the involving institutions *A* and *B* respectively for the *s*th variable.^25^ Then, we compute the $SI_{m}$ which is the average value of the similarity index of all the strategic variables with regards to the *m*th M &A.

Similarly to Altunbas and Marques-Ibanez (2008) we account for several indicators of the strategic relatedness of the merging firms. First, we consider those strategies related to their credit risk and loan-to-deposit characteristics. As far as the former is concerned, we measure credit risk strategy by the level of loan loss provisions divided by total assets. As far as the latter is concerned, we measure the banks’ loan and deposit profiles by the ratio of total loans to total customer deposits, which stands for a proxy for the use of relatively low-cost deposits in relation to the amount of loans outstanding. Additionally, we account for the banks broad balance sheet loan composition that is measured as the ratio of net loans to total assets, which takes into account the prominence of loans in banks’ total assets. Second, we consider the earnings diversification strategy, which is a broad product strategy that puts emphasis on other sources of income. i.e., non-interest income, off-balance sheet activities (OBS) apart from the traditional net interest revenues. We measure the former by the ratio of other operational revenue to total assets and the latter by the ratio of off-balance sheet activity to total assets. Third, we account for the cost controlling strategy that relates expenditure to revenues. We measure it by the cost to total income ratio. Fourth, we take into consideration the capital adequacy strategy that we measure it by the ratio of equity to total assets. Fifth, the liquidity risk strategy is considered, which is captured by the loans to assets ratio. Finally, we account for the technology and innovation strategy of banks, that we measure it by the ratio of other costs (i.e. total costs excluding interest, staff and other overhead payments) to total assets. Overall, the performance of the involving institutions is expected to deteriorate in the aftermath of a prospective consolidation activity the greater is the difference concerning the asset quality and the overall portfolio strategies among them.

## Empirical results

### Determination of the number of classes

One of the most important points in the estimation of the latent class models is the determination of the number of classes. A key method in the literature of the standard latent class models for identifying the number of regimes is the computation of an information criterion. The two most widely used statistics are the Akaike information criterion (AIC) and the Bayesian information criterion (BIC) or Schwarz criterion. The preferred model is the one with the lowest statistic.

The two statistics are computed as:

20 $$\begin{aligned}&AIC\left( K\right) =-LN\left( \sum \limits _{i=1}^{N}\sum \limits _{t=1}^{T_{i}} \left( \sum \limits _{k=1}^{K}p\left( k\shortmid i\right) \cdot \varepsilon _{it}^{2}\left( k\right) \right) \right) +\ln \left( \sum \limits _{i=1}^{N}T_{i}\right) +\frac{2\pi \left( K\right) }{\sum _{i=1}^{N}T_{i}} \end{aligned}$$

21 $$\begin{aligned}&BIC\left( K\right) =-2\cdot \ln LF\left( K\right) +\pi \left( K\right) \cdot \ln \left( \sum \limits _{i=1}^{N}T_{i}\right) \end{aligned}$$

where *K*, is the number of classes, $\pi \left( K\right)$ is the number of parameters to estimate for specification with *K* latent classes and $T_{i}$ is the number of observations for bank *i*.

Table 3a and b report the AIC and BIC values for the UK and Greek banking sectors respectively. Comparing a pooled model, that is, the baseline model as it was described in Sect. 3, which assumes homogenous production technology for all the financial institutions in the sample, that is, $$k=1$$, and a model with two different technological regimes, that is, $$ k=2$$, the values of both criteria indicate that the preferred model in both countries is the one with two classes.^26^

**Table 3:** (a) UK—Selection of the number of latent classes, (b) Greece—Selection of the number of latent classes

	No. of classes	No. of banks	No. of Param.	Log-Likelihood	AIC	BIC
(a)						
Pooled model	1	124	12	$-$ 456.9226	0.50998	0.54598
Latent class	2	73(1) 51(2)	28	$-$ 251.6265	0.30411	0.38811
(b)						
Pooled model	1	30	12	$-$ 4.211612	0.1247	0.28904
Latent class	2	17(1) 13(2)	28	90.97407	$-$ 0.48442	$-$ 0.10096

(a) Features stochastic frontier model estimations for 1 and 2 latent classes using 1856 observations and 124 UK financial institutions between 1988 and 2011. The preferred model is the one with the lowest AIC and BIC statistic

(b) Features stochastic frontier model estimations for 1 and 2 latent classes using 356 observations and 30 Greek financial institutions between 1993 and 2011. The preferred model is the one with the lowest AIC and BIC statistic

To illustrate this result, in Fig. 1a and b we plot the kernel density estimates of the variance of the residuals of inefficiency for both models for the UK and Greece, respectively. A leftward movement of the kernel in the second model with two technological regimes can easily be seen, implying that the inefficiency is removed when taking into account bank heterogeneity. Specifically, the sample is split by setting 17 and 73 banks in the first technological regime and 13 and 51 in the second one for Greece and the UK, respectively.

In order to check the sensitivity of the class size selection to inefficiency, we compute the average efficiency scores for each year, which are obtained by estimating models with one and two technological classes. These are reported in Table 4a for the UK and in Table 4b for Greece. One can see that the average efficiency monotonically increases with the number of classes. In turn, this suggests that if bank heterogeneity is not taken into account, this omission can lead to downward-biased efficiency score estimates.

In the “Appendix”, we examine which technological regime is the most efficient, we analyse the heterogeneous technologies using the determinants, we identify the financial institutions that belong to each technological class and we quote the alternative empirical strategy (Bos et al., 2010) that we adopt to estimate the latent class stochastic frontier framework in order to test the accuracy of our findings. Finally, we describe a series of robustness checks for the UK and Greek banking systems.

### Recent & prospective mergers and acquisitions

As a next step, we shed light on the various aspects of recent and potential M &A of UK and Greek banks. Our motivation steams from the significant changes that have been taking place since the summer of 2012 in the Greek banking sector.^27^ We endeavor to examine from an efficiency point of view whether the creation of the new bank will potentially move to the most efficient technological regime between the two existing ones,^28^ or even to a new and higher in terms of efficiency technological class that is created after the consolidation activity. In turn, we investigate, whether a prospective (i.e., before it is realised) M &A can increase the total factor productivity scores of the industry, resulting in larger efficiency synergies. In this way, we test for the first time in the literature M &A activity while accounting for different technological regimes. This is important, as after the onset of the global financial turmoil we witnessed many banks’ M &A, regardless of whether they were commercial, savings, co-operative, or real estate and mortgage banks. To this end, we investigate all the possible M &A combinations^29^ that could occur in the two banking sectors and we compute their respective efficiency gains or losses in real money terms. Our methodology can be applied to any micro-study and to any industry with either heterogeneous or homogenous production technologies, and in turn, help supervisory authorities and policymakers by providing in advance crucial information as far as the risk adjusted efficiency surplus or deficit that a consolidation activity may have are concerned.

**Table 4:** (a) UK—Average cost efficiency indexes with different number of classes, (b) Greece—Average cost efficiency indexes with different number of classes

Year	SFM with one Latent class	SFM with two Latent classes
(a)		
1988	0.48	0.68
1989	0.57	0.69
1990	0.49	0.68
1991	0.49	0.68
1992	0.58	0.67
1993	0.56	0.66
1994	0.58	0.65
1995	0.59	0.65
1996	0.61	0.66
1997	0.58	0.68
1998	0.61	0.7
1999	0.61	0.69
2000	0.58	0.66
2001	0.57	0.65
2002	0.57	0.64
2003	0.58	0.64
2004	0.61	0.65
2005	0.61	0.64
2006	0.61	0.64
2007	0.6	0.62
2008	0.6	0.62
2009	0.58	0.61
2010	0.56	0.59
2011	0.53	0.56
Total	0.57	0.65
(b)		
1993	0.63	0.69
1994	0.64	0.68
1995	0.66	0.69
1996	0.71	0.72
1997	0.68	0.76
1998	0.69	0.76
1999	0.67	0.73
2000	0.7	0.72
2001	0.71	0.73
2002	0.7	0.72
2003	0.7	0.71
2004	0.76	0.79
2005	0.73	0.82
2006	0.7	0.83
2007	0.72	0.86
2008	0.7	0.85
2009	0.69	0.84
2010	0.67	0.82
2011	0.64	0.79
Total	0.69	0.76

(a) Reports the average cost efficiency scores for each year of the UK banking industry, which are obtained by estimating stochastic frontier models with one and two technological classes

(b) Reports the average cost efficiency scores for each year of the Greek banking industry, which are obtained by estimating stochastic frontier models with one and two technological classes

**Table 5:** GREECE—M &As, Recapitalisation & Structure of the banking sector

Systemic banks	HFSF CAPITAL ENHANCEMENT (in millios of Euro in the end of 2013)	M &As	Year of the M &A activity
ALPHA BANK	4571	EMPORIKI	2012
EUROBANK	5839	NEW PROTON BANK, NEW TT-HELLENIC POSTBANK	2013 (Both financial institutions)
ETHNIKI BANK	8400	FBB, PROBANK	2013 (Both financial institutions)
PIRAEUS BANK	9756	ATE BANK, GENIKI BANK, MARFIN_EGNATIA, MILLENIUM	a. 2012 : ATE BANK and GENIKI Bank
			b. 2013: MARFIN- EGNATIA and MILLENIUM
*Remaining Banks*		*Type*	
ATTICA		Commercial	
AEGEAN		Commercial	
PANELLINIA		Commercial created by Co-operatives banks	
PANCRETAN		Co-operative	

This table reports detailed information about the recent wave of M &As where the ‘big-four’ of the Greek banking sector, i.e. ALPHA BANK, EUROBANK, ETHNIKI BANK and PIRAEUS BANK, were involved and resulted to the creation of the four ‘systemic’ banks. The table cites as well the total level of capital that the Hellenic Financial Stability Fund (HFSF) has injected in the four aforementioned systemic banks till the end of 2013, in order to facilitate both their soundness and ’the procedure of the consolidation activity that they were involved. Additionally, the table ‘presents the financial intermediaries’ and their business model that constitute the current structure of the Greek banking sector. As far as “ATE BANK, ’NEW PROTON BANK, NEW TT-HELLENIC POSTBANK, FBB and PROBANK” are concerned, only the ‘healthy’ part of assets and liabilities of those financial institutions was acquired. It should be noted that PIRAEUS BANK acquired in 2013 ‘CYPRUS BANK’ and ‘HELLENIC BANK’ as well, ’however due to unavailability of data we do not include these two cases. ‘ETHNIKI’ stands for the ’NATIONAL BANK OF GREECE’ while ‘MARFIN_EGNATIA’ stands for ’CYPRUS POPULAR BANK ‘(LAIKI BANK)’. ‘Finally, there are a few more ‘Co-operative’ type banks which we do not quote them as their aggregate market share is less than 2% in assets, deposits and loans of the whole banking sector

Before we continue with the analysis of the results, we highlight a discrepancy within the examination strategy of potential M &A of the two systems. For the UK banking sector, we select the 9 largest banks in terms of assets, deposits, and loans that belong to the most efficient technological regime (i.e. the first one) and the 11 largest from the second technological and less efficient group after we ensure that each of these 20 banks is not a subsidiary of the remaining 19. The methodology is as follows. We create every potential combination of M &A among the 9 and 11 respective banks in each regime. In this way, we are able to test whether the new bank would benefit from the M &A activity through a transition from a lesser to a more efficient class or would lose its efficiency level through the opposite move. Turning our attention to the Greek banking sector, we differentiate our empirical strategy due to the M &As took place. Specifically, we select all the remaining banks that have not been involved in the last wave of consolidation of the four systemic banks, and we create all potential combinations of M &A either among themselves or with one of the four cornerstones of the Greek economy. Additionally, we control for both single and multiple M &A by one banking institution. Last, regarding the four systemic banks, we examine both their recent and potential M &A in every possible combination (i.e. either one-by-one, two-by-two, etc. or by all the acquired banks together) to test what the bank’s regime/classification would be if it had not been involved in the last consolidation process. In Tables 6a and b we present all the cases of potential and recent/potential M &A activity for the UK and Greece, respectively, and their classification in the two different technological regimes.^30^ Moreover, both tables report information with respect to prospective gains or losses in real money values ($\pounds$/€) resulting from each hypothetical M &A that is quoted for both the pre-crisis period and the post-crisis period.^31^

**Table 6:** (a) UK—Hypothetical M &As Scenarios, (b) Greece—Hypothetical M &As Scenarios

		Class	dif[TC*CostInef]—Pre Crisis (M)	SI (%)	dif[TC*CostInef]—Post Crisis (M)	SI (%)	
(a)							
*Panel A: Potential M &As among banks in the* 2nd class							
_1	ALPHA-ACCESS	2	$-$ 296.24	11.38	$-$ 314.48	12.92	
_2	ALPHA-BEIRUT	1	195.52	5.47	228.19	6.03	
_3	ALPHA-CITIBANK	1	340.15	5.69	394.27	5.86	
_4	ALPHA-DBUKBANK	2	163.36	9.31	$-$ 217.85	11.18	
_5	ALPHA-EUROPE ARAB	2	242.72	9.18	$-$ 335.62	11.33	
_6	ALPHA-LEUMI	1	181.22	5.94	137.51	6.03	
_7	ALPHA-BAN OF NEW YORK	2	108.24	9.87	$-$ 134.19	10.07	
_8	ALPHA-PROGRESSIVE	1	157.51	4.62	119.61	5.81	
_9	ALPHA-SAINSBURY’S	2	$-$ 114.49	13.61	$-$ 151.73	14.03	
_10	ALPHA-UNION	2	$-$ 184.32	12.97	$-$ 207.91	13.42	
_11	LEUMI-ACCESS	2	$-$ 124.99	12.14	$-$ 177.48	12.76	
_12	LEUMI-BEIRUT	1	164.23	4.72	117.17	5.28	
_13	LEUMI-CITIBANK	1	296.45	4.88	354.16	6.23	
_14	LEUMI-DBUKBANK	2	$-$ 157.68	13.71	$-$ 185.95	14.24	
_15	LEUMI-EUROPE ARAB	2	191.07	7.93	$-$ 343.14	12.41	
_16	LEUMI-NEW_YORK	2	165.19	7.51	$-$ 243.06	12.94	
_17	LEUMI-PROGRESSIVE	1	124.95	4.79	218.51	5.17	
_18	LEUMI-SAINSBURY’S	2	$-$ 137.24	12.79	$-$ 361.59	12.96	
_19	LEUMI-UNION	2	$-$ 119.85	12.82	$-$ 177.56	13.06	
_20	BEIRUT-ACCESS	1	269.29	5.72	267.11	7.11	
_21	BEIRUT-CITIBANKJ	1	349.91	5.19	404.02	6.56	
_22	BEIRUT-DBUKBANK	2	162.04	8.11	$-$ 222.65	12.49	
_23	BEIRUT-EUROPE ARAB	2	224.32	8.96	$-$ 231.38	13.84	
_24	BEIRUT-NEWYORK	2	185.92	8.27	$-$ 231.19	13.97	
_25	BEIRUT-PROGRESSIVE	1	205.25	4.82	197.26	6.36	
_26	BEIRUT-SAINSBURY’S	2	$-$ 117.64	13.77	$-$ 242.94	14.29	
_27	BEIRUT-UNION	2	$-$ 347.62	14.27	$-$ 245.93	15.17	
_28	NEW_YORK-ACCESS	2	$-$ 293.16	15.73	$-$ 434.73	15.74	
_29	NEW_YORK-CITIBANK	2	394.62	8.63	$-$ 505.53	14.72	
_30	NEW_YORK-DBUKBANK	2	185.71	7.49	$-$ 454.96	13.17	
_31	NEW_YORK-EUROPE ARAB	2	188.97	7.86	$-$ 456.52	13.69	
_32	NEW_YORK-PROGRESSIVE	2	172.17	9.17	$-$ 438.88	14.46	
_33	NEW_YORK-SAIBURY’S	2	$-$ 219.46	14.31	$-$ 476.58	14.88	
_34	NEW_YORK-UNION	2	$-$ 138.41	16.74	$-$ 436.95	16.76	
_35	CITIBANK-ACCESS	2	$-$ 348.75	15.46	$-$ 409.38	15.53	
_36	CITIBANK-DBUKBANK	2	295.78	9.17	$-$ 353.56	14.53	
_37	CITIBANK-EUROPE ARAB	2	371.53	8.46	$-$ 430.37	14.81	
_38	CITIBANK-PROGRESSIVE	1	210.54	4.53	262.47	7.45	
_39	CITIBANK-SAINBURY’S	1	462.35	4.91	554.62	7.11	
_40	CITIBANK-UNION	2	$-$ 353.45	14.62	$-$ 412.97	15.19	
_41	DBUKBANK-EUROPE ARAB	2	138.76	8.36	$-$ 253.74	13.37	
_42	DBUKBANK-PROGRESSIVE	1	120.19	3.94	231.68	4.62	
_43	DBUKBANK-SAINSBURY’S	2	$-$ 139.33	12.76	$-$ 174.14	13.53	
_44	DBUKBANK-ACCESS	2	$-$ 109.46	12.63	$-$ 208.51	13.03	
_45	DBUKBANK-UNION	2	$-$ 120.95	14.39	$-$ 247.25	15.08	
_46	EUROPE ARAB-PROGRESSIVE	1	126.38	5.66	139.84	7.54	
_47	EUROPE ARAB-SAINSBURY’S	2	$-$ 148.65	14.11	$-$ 177.22	15.06	
_48	EUROPE ARAB-ACCESS	2	$-$ 123.48	15.36	$-$ 136.01	15.67	
_49	EUROPE ARAB-UNION	2	$-$ 129.46	15.87	$-$ 137.03	16.81	
_50	PROGRESSIVE-ACCESS	2	$-$ 88.65	14.83	$-$ 114.89	15.63	
_51	PROGRESSIVE-SAINSBURY’S	2	$-$ 115.67	16.15	$-$ 157.14	16.93	
_52	PROGRESSIVE-UNION	1	92.06	5.68	115.03	7.98	
_53	SAINSBURY’S -ACCESS	2	$-$ 127.62	13.79	$-$ 154.11	14.53	
_54	SAINSBURY’S -UNION	2	$-$ 139.65	14.54	$-$ 154.59	14.79	
_55	ACCESS-UNION	2	$-$ 74.66	13.83	$-$ 111.71	14.08	
*Panel B: Potential M &As among banks in* ***both classes***							
_1	BARCLAYS-ACCESS	2	$-$ 1426.5	19.43	$-$ 2278.41	19.65	
_2	BARCLAYS-ALPHA	1	4654.62	7.26	9339.09	7.87	
_3	BARCLAYS-LEUMI	1	4410.91	6.84	9968.47	7.09	
_4	BARCLAYS-BEIRUT	1	4588.26	6.71	9241.86	7.09	
_5	BARCLAYS-CITIBANK	2	$-$ 1604.96	18.83	$-$ 2765.78	19.73	
_6	BARCLAYS-DBUKBANK	2	4846.46	14.17	$-$ 9695.83	14.42	
_7	BARCLAYS-EUROPE ARAB	1	4521	6.83	8931.76	7.56	
_8	BARCLAYS-NEWYORK	2	4622.29	13.78	$-$ 9177.2	14.35	
_9	BARCLAYS-PROGRESSIVE	1	4355.07	7.23	8835.41	7.51	
_10	BARCLAYS-SAINSBURY’S	1	4297.16	5.34	9390.19	6.01	
_11	BARCLAYS-UNION	2	$-$ 4387.14	19.29	$-$ 17697.3	19.76	
_12	CO-OPERATIVE-ACCESS	2	$-$ 210.49	13.48	$-$ 456.09	13.53	
_13	CO-OPERATIVE-ALPHA	1	309.18	4.62	451.7	5.26	
_14	CO-OPRATIVE-BEIRUT	1	318.17	5.39	447.87	5.73	
_15	CO-OPERATIVE-CITIBANK	2	$-$ 589.16	12.81	$-$ 850.71	13.15	
_16	CO-OPERATIVE-DBUKBANK	2	$-$ 241.26	13.73	$-$ 476.8	14.28	
_17	CO-OPERATIVE-EUROPE ARAB	2	$-$ 229.15	12.27	$-$ 472.81	12.34	
_18	CO-OPERATIVE-LEUMI	1	337.06	4.33	461.73	5.61	
_19	CO-OPERATIVE-NEW_YORK	2	$-$ 406.26	13.46	$-$ 575.04	13.55	
_20	CO-OPERATIVE-PROGRESSIVE	2	$-$ 223.72	14.93	$-$ 455.81	15.47	
_21	CO-OPERATIVE-SAINBURY’S	2	$-$ 349.06	13.72	$-$ 599.33	13.82	
_22	CO-OPERATIVE-UNION	2	$-$ 281.05	12.08	$-$ 452.99	12.55	
_23	HABIB-ALPHA	1	36.66	3.27	54.17	3.59	
_24	HABIB-BEIRUT	1	21.69	3.86	34.78	4.35	
_25	HABIB-LEUMI	1	84.1	3.39	15.02	3.95	
_26	HABIB-ACCESS	2	$-$ 61.95	11.18	$-$ 90.79	11.71	
_27	HABIB-CITIBANK	2	$-$ 170.61	11.26	$-$ 139.46	11.64	
_28	HABIB-DBUKBANK	2	17.83	7.93	$-$ 25.2	11.34	
_29	HABIB-EUROPE ARAB	2	24.78	7.22	$-$ 34.64	11.02	
_30	HABIB-NEW_YORK	2	71.09	7.65	$-$ 134.82	11.48	
_31	HABIB-PROGRESSIVE	1	59.19	3.86	12.01	4.3	
_32	HABIB-SAINSBURY’S	2	$-$ 89.27	11.61	$-$ 149.8	11.76	
_33	HABIB-UNION	2	$-$ 61.56	11.83	$-$ 90.98	12.62	
_34	HSBC-ACCESS	2	$-$ 2754.56	18.66	$-$ 4166.57	19.08	
_35	HSBC-ALPHA	1	3903.56	7.24	7287.22	7.94	
_36	HSBC-BEIRUT	1	3852.11	6.41	7223.32	6.88	
_37	HSBC-CITIBANK	1	3925.25	6.58	7221.89	7.43	
_38	HSBC-DBUKBANK	2	4057.69	14.97	$-$ 4516.08	17.36	
_39	HSBC-EUROPE ARAB	2	3820.38	13.32	$-$ 4104.65	17.91	
_40	HSBC-LEUMI	1	3907.91	7.13	7305.28	7.83	
_41	HSBC-NEW_YORK	2	3975.68	14.71	$-$ 5382.91	17.26	
_42	HSBC-PROGRESSIVE	1	873.71	6.97	7284.19	7.46	
_43	HSBC-SAINSBURY’S	1	3904.56	7.04	7358.25	7.33	
_44	HSBC-UNION	2	$-$ 3894.17	19.43	$-$ 4189.95	20.02	
_45	LlOYDS-ACCESS	2	$-$ 4802.56	22.81	$-$ 6296.5	23.52	
_46	LlOYDS-ALPHA	1	5024.22	9.13	1834.3	10.65	
_47	LlOYDS-BEIRUT	1	4995.63	8.68	1736.95	8.72	
_48	LlOYDS-CITIBANK	2	4959.47	15.23	$-$ 6827.55	19.18	
_49	LlOYDS-DBUKBANK	2	5092.21	14.92	$-$ 6907.95	19.64	
_50	LlOYDS-EUROPE ARAB	2	4870.25	15.11	$-$ 6944.92	19.49	
_51	LlOYDS-LEUMI	2	4929.48	14.72	$-$ 6408.41	19.07	
_52	LlOYDS-NEW_YORK	2	4949.44	14.17	$-$ 6392.38	19.55	
_53	LlOYDS-PROGRESSIVE	2	4855.67	15.21	$-$ 6128.18	20.01	
_54	LlOYDS-SAINSBURY’S	2	$-$ 2825.17	21.45	$-$ 6517.86	21.73	
_55	LlOYDS-UNION	2	$-$ 1719.06	22.08	$-$ 6582.57	22.44	
_56	NATIONWIDE-ACCESS	2	$-$ 420.92	16.71	$-$ 872.27	17.84	
_57	NATIONWIDE-ALPHA	1	555.71	6.13	947.98	6.59	
_58	NATIONWIDE-BEIRUT	1	503.36	6.38	868.58	7.07	
_59	NATIONWIDE-CITIBANK	1	816.06	6.41	1257.86	7.04	
_60	NATIONWIDE-DBUKBANK	2	628.03	12.66	$-$ 1056.14	17.58	
_61	NATIONWIDE-EUROPE ARAB	1	770.59	6.85	1268.99	7.22	
_62	NATIONWIDE-LEUMI	1	625.32	6.39	1055.29	6.55	
_63	NATIONWIDE-NEW YORK	1	815.28	6.74	1368.67	7.59	
_64	NATIONWIDE-PROGRESSIVE	1	621.33	6.53	1050.01	7.48	
_65	NATIONWIDE-SAINSBURY’S	1	702.59	7.09	1256.84	7.92	
_66	NATIONWIDE-UNION	1	527.65	6.11	1092.62	6.75	
_67	RBS-ACCESS	2	$-$ 8432.48	23.18	$-$ 7921.57	23.99	
_68	RBS-ALPHA	2	2878.13	17.14	$-$ 7388.76	23.08	
_69	RBS-BEIRUT	1	2847.86	9.13	1765.73	9.94	
_70	RBS-CITIBANK	2	514.63	16.85	$-$ 6476.14	22.48	
_71	RBS-DBUKBANK	2	$-$ 2168.71	22.93	$-$ 7389.31	23.19	
_72	RBS-EUROPE ARAB	2	$-$ 2379.19	22.71	$-$ 6816.68	23.45	
_73	RBS-LEUMI	1	2879.84	9.42	1421.2	9.96	
_74	RBS-NEW_YORK	2	2745.87	16.43	$-$ 6976.79	22.45	
_75	RBS-PROGRESSIVE	1	2672.64	9.68	1967.75	10.04	
_76	RBS-SAINSBURY’S	2	$-$ 1716.27	22.82	$-$ 7151.32	23.18	
_77	RBS-UNION	2	$-$ 1268.37	23.46	$-$ 7314.78	24.43	
_78	SANTANDER-ACCESS	2	$-$ 618.56	15.83	$-$ 2001.99	16.03	
_79	SANTANDER-ALPHA	1	533.48	7.08	601.75	7.73	
_80	SANTANDER-BEIRUT	1	1313.38	7.36	1999.53	7.51	
_81	SANTANDER-CITIBANK	2	$-$ 733.92	15.91	$-$ 782.01	15.96	
_82	SANTANDER-DBUKBANK	2	1285.36	13.76	$-$ 1973.6	17.33	
_83	SANTANDER-EUROPE ARAB	2	1339.35	14.12	$-$ 2014.58	17.21	
_84	SANTANDER-LEUMI	1	1161.88	7.72	1794.1	8.11	
_85	SANTANDER-NEW_YORK	2	1262.84	13.93	$-$ 1959.15	17.67	
_86	SANTANDER-PROGRESSIVE	2	1155.13	13.18	$-$ 1784.74	17.82	
_87	SANTANDER-SAINSBURY’S	2	$-$ 1272.17	16.87	$-$ 2159.51	17.47	
_88	SANTANDER-UNION	2	$-$ 859.35	17.15	$-$ 2010.11	18.19	
_89	STANDARD-ACCESS	2	$-$ 1269.31	17.38	$-$ 6497.3	17.63	
_90	STANDARD-ALPHA	1	1406.69	7.67	1594.5	8.34	
_91	STANDARD-BEIRUT	1	1396.94	7.93	1579.45	7.99	
_92	STANDARD-CITIBANK	2	1325.61	15.33	$-$ 2988.39	18.09	
_93	STANDARD-DBUKBANK	2	1452.92	15.92	$-$ 2708.24	18.49	
_94	STANDARD-EUROPE ARAB	2	1385.06	15.49	$-$ 2441.2	18.66	
_95	STANDARD-LEUMI	2	1281.66	15.91	$-$ 2304.23	18.73	
_96	STANDARD-NEW_YORK	2	1459.35	15.07	$-$ 2651.65	16.24	
_97	STANDARD-PROGRESSIVE	1	1270.46	7.34	1263.78	7.78	
_98	STANDARD-SAINSBURY’S	1	1462.72	7.28	1693.62	7.48	
_99	STANDARD-UNION	2	$-$ 1346.16	17.53	$-$ 2583.71	18.16	

		CLASS	dif[TC*CostInef]—Pre Crisis (M)	SI (%)	dif[TC*CostInef]—Post Crisis (M)	SI (%)	*HFSF*
(b)							
*Panel A: Recent - M &As*							
_1	ALPHA-EMPORIKI	1	108.43	6.76	271.79	8.83	–
_2	EUROBANK-PROTON-TT_HELLENIC	2	$-$ 27.54	11.68	$-$ 144.85	13.47	–
_3	ETHNIKI-FFB-PROBANK	1	108.49	8.43	322.38	11.43	–
_4	PIRAEUS-ATE-GENIKI-MARFIN_EGNATIA-MILLENIUM	2	$-$ 61.53	14.63	$-$ 204.04	16.34	–
*Panel B: Recent (Potential) - M &As*							
_1	EUROBANK-PROTON	2	21.46	8.44	$-$ 94.01	11.05	50.84
_2	EUROBANK-TT_HELLENIC	1	151.72	6.69	105.3	8.63	250.2
_3	ETHNIKI-FBB	1	74.06	7.51	212.82	9.32	$-$ 109.6
_4	ETHNIKI-PROBANK	1	59.6	7.32	184.74	9.21	$-$ 137.6
_5	PIRAEUS-ATE	2	17.56	9.37	$-$ 129.36	12.56	74.68
_6	PIRAEUS-MARFIN_EGNATIA	1	141.96	7.68	82.21	10.14	286.3
_7	PIRAEUS-MILLENIUM	1	120.45	7.55	47.82	9.56	251.9
_8	PIRAEUS-GENIKI	2	58.83	9.78	$-$ 77.58	12.94	126.5
_9	PIRAEUS-ATE-GENIKI	2	7.55	10.27	$-$ 119.37	12.69	84.67
_10	PIRAEUS-MILLENIUM-GENIKI	2	76.56	10.59	$-$ 74.58	12.92	129.5
_11	PIRAEUS-MARFIN_EGNATIA-GENIKI	2	38.79	10.38	$-$ 82.35	12.59	121.7
_12	PIRAEUS-MILLENIUM-MARFIN_EGANTIA	1	170.69	8.83	5.04	10.7	209.1
_13	PIRAEUS-MILLENIUM-ATE	2	17.15	10.64	$-$ 113.03	12.33	91.01
_14	PIRAEUS-MARFIN_EGANTIA-ATE	2	49.02	10.89	$-$ 118.49	13.05	85.55
_15	PIRAEUS-ATE-GENIKI-MARFIN_EGANTIA	2	28.26	12.46	$-$ 121.39	14.34	82.65
_16	PIRAEUS-ATE-GENIKI-MILLENIUM	2	13.39	12.73	$-$ 97.49	15.21	106.6
_17	PIRAEUS-GENIKI-MILLENIUM-MARFIN_EGNATIA	2	28.91	13.64	$-$ 104.13	15.39	99.91
*Panel C: Potential - M &As*							
_1	ALPHA-ATTICA	1	35.55	6.91	132.58	9.17	$-$ 139.2
_2	ALPHA-AEGEAN	1	37.37	7.13	141.96	8.76	$-$ 129.8
_3	ALPHA-PANELLINIA	1	23.29	7.27	106.87	8.88	$-$ 164.9
_4	ALPHA-PANCRETAN	1	27.71	8.94	101.83	10.76	$-$ 170
_5	ALPHA-EMPORIKI-ATTICA	2	$-$ 54.89	10.17	$-$ 51.34	11.31	$-$ 323.1
_6	ALPHA-EMPORIKI-AEGEAN	2	104.42	9.26	$-$ 25.84	11.15	$-$ 297.6
_7	ALPHA-EMPORIKI-PANELLINIA	1	118.27	8.26	238.9	10.09	$-$ 32.89
_8	ALPHA-EMPORIKI-PANCRETAN	1	112.89	8.43	292.5	10.75	20.71
_9	ALPHA-EMPORIKI-ATTICA-AEGEAN	2	111.74	11.06	$-$ 24.66	13.31	$-$ 296.5
_10	ALPHA-EMPORIKI-ATTICA-PANELLINIA	2	150.34	10.89	$-$ 71.11	13.01	$-$ 342.9
_11	ALPHA-EMPORIKI-ATTICA-PANCRETAN	2	185.59	10.77	$-$ 82.24	13.21	$-$ 354
_12	ALPHA-EMPORIKI-AEGEAN-PANNELINIA	1	194.83	9.84	347.79	12.07	76
_13	ALPHA-EMPORIKI-AEGEAN-PANCRETAN	1	174.53	9.72	352.63	11.49	80.84
_14	ALPHA-EMPORIKI-PANELLINIA-PANCRETAN	2	121.23	10.55	$-$ 100.09	13.69	$-$ 371.9
_15	ALPHA-EMPORIKI-ATTICA-AEGEAN-PANELLINIA	2	200.15	12.81	$-$ 125.73	15.24	$-$ 397.5
_16	ALPHA-EMPORIKI-ATTICA-AEGEAN-PANCRETAN	2	240.67	12.75	$-$ 145.74	15.22	$-$ 417.5
_17	ALPHA-EMPORIKI-ATTICA-PANELLINIA-PANCRETAN	2	236.75	12.27	$-$ 84.27	14.26	$-$ 356.1
_18	ALPHA-EMPORIKI-AEGEAN-PANELLINIA-PANCRETAN	2	$-$ 143.72	12.09	$-$ 159.55	13.69	$-$ 431.3
_19	ALPHA-EMPORIKI-ATTICA-AEGEAN-PANELLINIA-PANCRETAN	2	$-$ 162.92	13.87	$-$ 194.55	14.43	$-$ 466.3
_20	EUROBANK-ATTICA	1	143.94	7.92	110.72	10.29	255.6
_21	EUROBANK-AEGEAN	1	106.82	8.09	106.04	10.41	250.9
_22	EUROBANK-PANELLINIA	1	115.97	8.53	94.15	10.21	239
_23	EUROBANK-PANCRETAN	1	114.84	9.92	85.35	11.87	230.2
_24	EUROBANK-PROTON-TT_HELLENIC-ATTICA	2	$-$ 28.44	12.72	$-$ 184.7	14.57	$-$ 39.85
_25	EUROBANK-PROTON-TT_HELLENIC-AEGEAN	2	$-$ 4.63	12.93	$-$ 133.83	15.41	11.02
_26	EUROBANK-PROTON-TT_HELLENIC-PANELLINIA	2	$-$ 8.26	11.64	$-$ 149.05	15.19	$-$ 4.2
_27	EUROBANK-PROTON-TT_HELLENIC-PANCRETAN	1	149.06	11.76	112.87	13.61	257.7
_28	EUROBANK-PROTON-TT_HELLENIC-ATTICA-AEGEAN	2	23.58	14.39	$-$ -234.85	16.44	$-$ 90
_29	EUROBANK-PROTON_TT-HELLENIC-ATTICA-PANELLINIA	2	3.52	13.97	$-$ 263.97	16.18	$-$ 119.1
_30	EUROBANK-PROTON-TT_HELLENIC-ATTICA-PANCRETAN	2	17.19	13.84	$-$ 216.79	15.66	$-$ 71.94
_31	EUROBANK-PROTON-TT_HELLENIC-AEGEAN-PANELLINIA	2	$-$ 17.94	15.78	$-$ 253.69	15.39	$-$ 108.8
_32	EUROBANK-PROTON-TT_HELLENIC-AEGEAN-PANCRETAN	2	0.49	13.69	$-$ 246.95	15.52	$-$ 102.1
_33	EUROBANK-PROTON_TT-HELLENIC-PANELLINIA-PANCRETAN	2	$-$ 7.02	16.21	$-$ 278.79	15.09	$-$ 133.9
_34	EUROBANK-PROTON-TT_HELLENIC-ATTICA-AEGEAN-PANELLINIA	2	39.83	15.08	$-$ 152.68	16.79	$-$ 7.83
_35	EUROBANK-PROTON-TT_HELLENIC-ATTICA-AEGEAN-PANCRETAN	2	44.48	15.16	$-$ 123.59	17.46	21.26
_36	EUROBANK-PROTON-TT_HELLENIC-ATTICA-PANELLINIA-PANCRETAN	2	27.63	14.43	$-$ 160.04	16.23	$-$ 15.19
_37	EUROBANK-PROTON-TT_HELLENIC-AEGEAN-PANELLINIA-PANCRETAN	2	30.81	14.52	$-$ 187.46	16.87	$-$ 42.61
_38	EUROBANK-PROTON-TT_HELLENIC-ATTICA-AEGEAN-PANELLINIA-PANCRETAN	*2*	$-$ 8.82	16.94	$-$ 219.8	18.21	$-$ 74.95
_39	ETHNIKI-ATTICA	1	64.22	7.16	267.96	9.62	$-$ 54.42
_40	ETHNIKI-AEGEAN	1	52.65	7.89	243.18	9.88	$-$ 79.2
_41	ETHNIKI-PANELLINIA	1	37.71	7.53	212.2	10.17	$-$ 110.2
_42	ETHNIKI-PANCREATAN	1	29.99	8.51	182.7	11.15	$-$ 139.7
_43	ETHNIKI-FFB-PROBANK-ATTICA	1	157.17	9.83	383.19	12.77	60.81
_44	ETHNIKI-FFB-PROBANK-AEGEAN	2	142.7	10.08	$-$ 74.85	13.11	$-$ 397.2
_45	ETHNIKI-FFB-PROBANK-PANELLINIA	2	117.65	10.16	$-$ 63.09	13.09	$-$ 385.5
_46	ETHNIKI-FFB-PROBANK-PANCRETAN	1	106.86	9.51	260.92	11.74	$-$ 61.46
_47	ETHNIKI-FFB-PROBANK-ATTICA-AEGEAN	2	113.17	11.67	$-$ 172.48	13.07	$-$ 494.9
_48	ETHNIKI-FFB-PROBANK-ATTICA-PANELLINIA	1	109.41	10.24	181.17	12.16	$-$ 141.2
_49	ETHNIKI-FFB-PROBANK-ATTICA-PANCRETAN	1	124.26	10.37	198.17	11.48	$-$ 124.2
_50	ETHNIKI-FFB-PROBANK-AEGEAN-PANELLINIA	2	150.14	11.49	$-$ 150.51	13.84	$-$ 472.9
_51	ETHNIKI-FFB-PROBANK-AEGEAN-PANCRETAN	2	117.03	11.71	$-$ 92.52	13.57	$-$ 414.9
_52	ETHNIKI-FFB-PROBANK-PANELLINIA-PANCRETAN	1	93.4	10.07	122.83	12.02	$-$ 199.6
_53	ETHNIKI-FFB-PROBANK-ATTICA-AEGEAN-PANELLINIA	2	121.51	13.76	$-$ 178.59	14.14	$-$ 501
_54	ETHNIKI-FFB-PROBANK-ATTICA-AEGEAN-PANCRETAN	2	141.94	13.91	$-$ 189.22	14.83	$-$ 511.6
_55	ETHNIKI-FFB-PROBANK-ATTICA-PANELLINIA-PANCRETAN	1	130.64	12.43	284.69	13.25	$-$ 37.69
_56	ETHNIKI-FFB-PROBANK-AEGEAN-PANELLINIA-PANCRETAN	2	104.85	13.22	$-$ 139.91	14.44	$-$ 462.3
_57	ETHNIKI-FFB-PROBANK-ATTICA-AEGEAN-PANELLINIA-PANCRETAN	2	136.64	14.08	$-$ 187.61	15.13	$-$ 510
_58	PIRAEUS-ATTICA	1	13.39	8.27	141.42	10.66	345.5
_59	PIRAEUS-AEGEAN	1	28.91	8.83	113.64	10.97	317.7
_60	PIRAEUS-PANELLINIA	1	153.7	9.06	87.84	10.75	291.9
_61	PIREAUS-PANCRETAN	1	142.32	10.41	92.43	12.47	296.5
_62	PIRAEUS-ATE-MARFIN_EGNATIA-MILLENIUM-GENIKI-ATTICA	2	130.2	15.17	$-$ 276.91	17.99	$-$ 72.87
_63	PIRAEUS-ATE-MARFIN_EGNATIA-MILLENIUM-GENIKI-AEGEAN	2	126.73	15.12	$-$ 234.37	17.72	$-$ 30.33
_64	PIRAEUS-ATE-MARFIN_EGNATIA-MILLENIUM-GENIKI-PANELLINIA	2	$-$ 83.96	18.43	$-$ 206.35	18.25	$-$ 2.31
_65	PIRAEUS-ATE-MARFIN_EGNATIA-MILLENIUM-GENIKI-PANCRETAN	2	$-$ 56.96	18.18	$-$ 192.34	17.86	11.7
_66	PIRAEUS-ATE-MARFIN_EGNATIA-MILLENIUM-GENIKI-ATTICA-AEGEAN	1	64.97	16.79	146.45	18.9	350.5
_67	PIRAEUS-ATE-MARFIN_EGNATIA-MILLENIUM-GENIKI-ATTICA-PANELLINIA	2	$-$ 59.43	18.34	$-$ 290.94	19.62	$-$ 86.9
_68	PIRAEUS-ATE-MARFIN_EGNATIA-MILLENIUM-GENIKI-ATTICA-PANCRETAN	1	183.91	15.98	123.38	16.73	327.4
_69	PIRAEUS-ATE-MARFIN_EGNATIA-MILLENIUM-GENIKI-AEGEAN-PANELLINIA	2	$-$ 89.03	18.67	$-$ 253.8	20.06	$-$ 49.76
_70	PIRAEUS-ATE-MARFIN_EGNATIA-MILLENIUM-GENIKI-AEGEAN-PANCRETAN	2	175.9	17.09	$-$ 73.29	19.45	130.8
_71	PIRAEUS-ATE-MARFIN_EGNATIA-MILLENIUM-GENIKI-PANELLINIA-PANCRETAN	2	$-$ 78.56	18.13	$-$ 281.19	19.16	$-$ 77.15
_72	PIRAEUS-ATE-MARFIN_EGNATIA-MILLENIUM-GENIKI-ATTICA-AEGEAN-PANELLINIA	2	148.83	17.93	$-$ 296.69	20.43	$-$ 92.65
_73	PIRAEUS-ATE-MARFIN_EGNATIA-MILLENIUM-GENIKI-ATTICA-AEGEAN-PANCRETAN	2	$-$ 94.27	18.76	$-$ 273.29	20.48	$-$ 69.25
_74	PIRAEUS-ATE-MARFIN_EGNATIA-MILLENIUM-GENIKI-ATTICA-PANELLINIA-PANCRETAN	2	$-$ 129.68	18.64	$-$ 336.24	20.63	$-$ 132.2
_75	PIRAEUS-ATE-MARFIN_EGNATIA-MILLENIUM-GENIKI-AEGEAN-PANELLINIA-PANCRETAN	2	$-$ 109.8	18.55	$-$ 307.03	20.07	$-$ 102.99
_76	PIRAEUS-ATE-MARFIN_EGNATIA-MILLENIUM-GENIKI-ATTICA-AEGEAN-PANELLINIA-PANCRETAN	2	$-$ 157.86	19.83	$-$ 358.94	21.73	$-$ 154.9
_77	ATTIKA-AEGEAN	2	$-$ 3.45	5.49	$-$ 28.84	7.99	–
_78	ATTICA-PANELLINIA	2	7.16	5.17	$-$ 1.93	7.37	–
_79	ATTICA-PANCRETAN	2	$-$ 2.4	6.97	$-$ 19.64	7.52	–
_80	AEGEAN-PANELLINIA	2	4.7	5.12	$-$ 4.92	6.72	–
_81	AEGEAN-PANCRETAN	1	4.58	4.16	8.64	5.13	–
_82	PANELLINIA-PANCRETAN	2	2.53	7.22	$-$ 1.23	8.77	–
_83	ATTICA-AEGEAN-PANELLINIA	2	$-$ 13.61	7.48	$-$ 62.75	9.34	–
_84	ATTICA-AEGEAN-PANCRETAN	1	1.13	6.28	19.62	7.39	–
_85	ATTICA-PANELLINIA-PANCRETAN	2	$-$ 7.17	7.05	$-$ 32.08	9.07	–
_86	AEGEAN-PANELLINIA-PANCRETAN	2	2.69	7.14	$-$ 1.38	8.76	–
_87	ATTICA-AEGEAN-PANELLINIA-PANCRETAN	1	86.45	7.81	157.39	7.83	–

(a) Reports all the prospective scenarios of M &As among 20 UK financial institutions and the classification of the ‘new’ financial entity into the two latent technological classes according to the regime membership determinants described in Table 2a. Specifically, we select the nine most important financial intermediaries in terms of assets, deposits and loans that belong to the most efficient technological regime (i.e. the first one) and the eleven most important from the second technologically and less efficient class after we ensure that each of these latter twenty banks is not a subsidiary of the remaining nineteen. Panel A in the first column presents all possible combinations of consolidation between those financial institutions that belong to the second and less efficient technological class, while Panel B in the first column reposts all possible combinations of consolidation between those financial institutions that belong to different technological regime. ‘difTotal Cost * Cost Ineff’ measures the difference of the total cost associated with the level of cost inefficiency between the individuals ones (A$+$B) and the prospective financial institution (AB) and indicates prospective gains (positive sign) or losses (negative sign) in real money values ($\pounds$) resulting from each hypothetical M &A that is quoted for both the ‘pre’ crisis and the ‘post’ crisis period. ‘SI’ represents the average value of the similarity index of all the strategic variables regarding each M &A. ‘M’ stands for million

(b) Reports all the prospective scenarios of M &As among all the Greek financial institutions and the classification of the ‘new’ financial entity into the two latent technological classes according to the regime membership determinants described in Table 2b. Panel A in the first column entitled ‘Recent’ consists of all consolidation activities that took place recently and created the four so-called ‘Systemic’ banks (ALPHA, ETHNIKI, EUROBANK, PIRAEUS). Panel B in the first column entitled ‘Recent (Potential)’ consists of all possible combinations of consolidation between the ‘big four’ of the Greek banking sector and the institutions that they finally were absorbed by them and altogether formedtheir systemic nature. We approach each one of these cases in both categories as a prospective M &A scenario in the economy, since our sample is dated up to 2011 and the recentconsolidation wave took place in 2012 and 2013. Panel C in the first column entitled ‘Potential’ reports all possible combinations of consolidation between the four major banks of the Greek economy, before and after they got involved into the recent wave of M &As, and the four remaining banking institutions namely, Attica bank, Aegean bank, Panellinia bank and Pancretan. The table presents all possible combinations of consolidation among those four remaining banks (i.e. only non-systemic banks)and the classification of the new financial entity as well. ‘dif[TC*CostInef]’ measures the difference of the total cost associated with the level of cost inefficiency between the ’individuals ones (A$+$B) and the prospective financial institution (AB) and indicates prospective gains (positive sign) or losses (negative sign) in real money values (€) resulting from each hypothetical M &A that is quoted for both the ‘pre’ crisis and the ‘post’ crisis period. ‘SI’ represents the average value of the similarity index of all the strategic variables regarding each M &A. ‘HFSF’ indicates prospective gains (negative sign) or losses (positive sign) in real money values (€) for the Hellenic Financial Stability Fund (HFSF) and consequently for the Greek Economy and its tax payers in general, that result from each hypothetical M &A activity where each one of the four ‘Systemic’ banks could have been involved into, instead of the ‘Recent’ wave of M &As that was actually realised. All gains and losses with respect to ‘HFSF’ refer to the ‘post’ crisis period since the HFSF did not exist in the ‘pre’ crisis era. ‘M’ stands for million

#### UK—prospective M &As

Here, we focus our analysis on the UK banking sector and its potential consolidation wave. Table 6a shows the results for all potential M &A activity regarding the 20 (9 in the first technological regime and 11 in the second) largest banks in terms of assets, loans, and deposits at the end of our sample period. Specifically, we account for every potential combination of M &A among those financial institutions that belong to different classes and among those that are all found ex-ante in the second regime to examine whether a specific consolidation activity can result in the transition of the new bank in the higher technological regime (i.e. the first one) in terms of efficiency.^32^

As far as the category of potential M &A among the banks that belong in the two different regimes is concerned, in approximately $40\%$ of the cases the new financial institution will be classed in the first and most efficient technological regime (see Panel B). It is noteworthy that $20\%$ of these potential M &A cases involve a building society, namely Nationwide, and not a bank. Additionally, our results indicate that two of the big four of the UK banking sector, namely Barclays and HSBC, account for a bit less than a quarter of the potential M &A cases that result in enhanced efficiency, whereas the remaining two large UK banks (RBS and Lloyds) account for just $12\%$ and $8\%$, respectively, of those potential M &A that create a financial institution with a higher efficiency level than before. This might reflect the calamitous impact of the financial crisis on the latter pair of banks, which resulted in significant financial assistance by the UK government with the aim of avoiding the collapse of both banks.^33^ Regarding the banks that belonged to the second group before they were involved in M &A activity, we notice that in $75\%$ of the cases, three banks and one building society are found to create a financial institution that belongs to the most efficient class following their consolidation with their peers from the first technological regimes.

We now examine the potential combinations of consolidation among the financial institutions that belong in the second technological regime. Contrary to the previous picture, we infer that approximately in only $25\%$ of the overall cases we find the new bank to be classified in the first regime (see Panel A). What is interesting is that the aforementioned three banks and the one building society account once again for two-thirds of the overall cases where we experience a transition towards a more efficient technological regime. Last, our results show that the largest financial institution among those that belong to the second regime, would experience a transition to the first and more efficient technological class if it merged with one of either the big four of the UK banking system or with Santander, or Standard Chartered.

Finally yet importantly, we are interested to quantify the efficiency benefits or losses in real money terms of a potential M &A activity. Thereby, we measure for each consolidation case, the difference of the total cost associated with the level of cost inefficiency of the involving financial institutions between their pre and post M &A status. It is noteworthy that in the vast majority of cases, if the new financial institution is classified in the first and more efficient regime, regardless of whether the emerged bank consisted of institutions that were allocated either to different technological classes or to the same one, there will be a cost reduction (see positive sign in the respective columns in Table 6a), that is, an economic gain in real money terms in both periods around the crisis. This is of extreme importance, especially for RBS and Lloyds, as a prospective consolidation activity of each one of those two banks with some specific financial institutions could lead to cost benefits and thus to the alleviation of the taxpayers’ burden, as the UK government’s bailout program, where it partially nationalized both banks, could have been smaller. On the contrary, when the consolidated institution is allocated to the second technological regime, the results are mixed with respect to the pre-crisis period. Whereas in most of the cases regarding the aftermath of the crisis,^34^ it is evident that there is a deterioration with respect to the cost, highlighting the detrimental negative impact of the last financial turmoil on the cost efficiency of those institutions.

#### Greece—recent M &As

One of the most substantial finding as far as the Greek banking sector is concerned is that two out of the four newly designed engines to promote the Greek economic recovery, namely Eurobank and Piraeus, are found after their series of acquisitions to be in the less efficient technological class as opposed to the other two, Alpha Bank and Ethniki Bank, which despite their recent acquisitions still belong to the first technological regime (see Table 6b, Panel A). On the one hand, it seems that if Eurobank had absorbed only TT-Hellenic Postbank, the new bank would have placed in the first and higher efficiency regime, whereas the acquisition of only Proton Bank (without TT-Hellenic Postbank) would have deteriorated Eurobank’s position before any M &A activity had occurred (see Panel B). On the other hand, it may be easier to comprehend the case of Piraeus Bank, as it is involved in the largest consolidation activity that may affect its efficiency levels. In order to provide a more thorough explanation, we look at each one of Piraeus Bank’s acquisitions separately and gradually add to them another financial institution from the list of banks that were absorbed in the end. Table 6b (Panel B) demonstrates the results. It is noteworthy that only two banks, namely Marfin Egnatia Bank and Millenium Bank, after being acquired by Piraeus Bank either individually or simultaneously, would have led to a newly created bank that would have been allocated to the most efficient technological class. On the contrary we find evidence that every combination of banking institutions regarding the potential M &A of Pireaus bank with ATE Bank and/or Geniki Bank with or without the presence of Marfin Egnatia Bank and Millenium Bank places the new bank in the second and less efficient regime.^35^ The last points cast major doubt on the ability of specific banks’ M &A in the last wave of consolidation in Greece to generate and pass on merger-specific synergies to the economy. Consequently, concerns are raised about the decisions of the policymakers and about involving banks in the selection process about which financial institution will be the acquirer and which one will be the target in terms of the resulting economic benefit of the consolidation process. However, we confirm the concerns of the officials regarding the cancelled attempt at consolidation of two of the four big banks, namely Ethiki and Eurobank, as we find a potential M &A entity among them in the less efficient technological regime.

#### Greece—prospective M &As

Turning our attention now to potential M &A between the four major banks of the new era of the Greek economy and the four remaining banking institutions, namely Attica Bank, Aegean Bank, Panellinia Bank, and Pancretan Co-operative Bank,^36^ we acquire some insightful outcomes. We examine all potential combinations of consolidation between the last four banking institutions, which are equally split among the two technological regimes, with or without the four systemic banks and before and after their recent acquiring activity (see Panel C). It is noteworthy to see that all potential M &A of each of the four remaining banks with each of the systemic banks before they got involved in the last consolidation wave would have resulted in the new bank being classed into the first technological regime. This would be even more important for Attica Bank and Panellinia Bank as it would upgrade their efficiency levels because they both belong to the second class.

Shedding light on all future possible combinations of M &A between the remaining four banks and the four systemic banks reveals that the two co-operative banks (Pancretan and Panellinia) and Aegean Bank create combinations of M &As where most of the time the new bank is found to be classified in the first technological regime. The first systemic bank, Alpha Bank, in the aftermath of Emporiki’s acquisition, seems to create four out of fifteen of its overall potential combinations of M &A that are found to exhibit high efficiency levels, that is, that belong in the first technological class. These four prospective scenarios are constituted of the two co-operative banks and in two cases of the Aegean bank as well. We find similar results regarding Ethniki Bank (and FFB Bank and Probank as well) and its potential combinations of consolidation with non-systemic banks. The estimation results show that in $30\%$ of the overall cases, the new bank will be allocated in the first and most efficient technological class and thus enhance its level of cost efficiency due to the prospective consolidation activity. All the cases include Pancreatan Bank. Nevertheless, there is a high frequency of the appearance of both Attica Bank and Panellinia Bank. This is of extreme importance, as those two financial institutions are initially found in the lower technologically efficient class, and it seems that their efficiency levels would have been enhanced after the specific prospective M &A. On the contrary, only approximately $7\%$ of the potential combinations of the current structure of Eurobank (that is, it has already absorbed both the New Proton Bank and the New TT-Hellenic Postbank) with the four non-systemic banks creates a new bank that will have higher levels of efficiency. This will consist of a potential M &A between the new systemic Eurobank and Pancretan Bank. The remaining systemic bank, Pireaus Bank (with ATE Bank, Geniki Bank, Marfin Egnatia Bank and Millenium Bank), creates twice as many M &A cases than Eurobank that are in the first technological regime (i.e. which have enhanced efficiency levels). This consists of potential combinations of M &As among the new systemic Piraeus Bank either with Attica Bank or with Attica Bank and one of Aegean Bank or Pancretan-Cooperative Bank. All these results strengthen our initial and main finding that two out of the four systemic banks classified in the highest technological class in terms of efficiency are the ones that create potential combinations of consolidation whose efficiency is enhanced after the potential M &A activity.

As a last exercise, we examine the non-systemic banks and their potential interactions. We can infer that $30\%$ of the overall potential combinations of those four banking institutions is classified in the first technological regime. All the successful (i.e. enhanced efficiency after the consolidation process) combinations consist of either Aegean Bank or Pancretan Bank with either Attica Bank or with the combination of both Attica Bank and Panellinia Bank together. This outcome is of great interest as both Attica Bank and Panellinia Bank belong to the second technological regime. Thus, based on the empirical evidence, it seems that both can achieve higher efficiency levels after a potential consolidation with either Aegean Bank or Pancretan Bank. In turn, our results indicate that there are still considerable economies of scale for the smaller financial institutions in Greece that need to be exploited.

Additionally, as in the case of the UK banking sector, on all the occasions where the consolidated financial entity is classified in the higher technological regime in terms of efficiency, it would lead to significant cost reductions in real money terms in both the pre-crisis period and in the post-crisis period. On the contrary, regarding those new financial institutions allocated to the second technological regime, in most cases and for both distinct economic periods they do not create any beneficial cost efficiency synergies (see negative sign in the respective columns in Table 6b). Notable exceptions from the previous category (i.e. the new bank belongs to the second regime but the consolidation process leads to a cost reduction) are a few potential M &A cases created by Alpha Bank and Ethniki bank in either their *pre* or *post* systemic formation. This is in line with our concerns about whether two of the four cornerstones of the restructured Greek banking sector (i.e. Eurobank and Piraeus bank) following the last wave of M &A could benefit the economy. Last, in order to be more precise on the extracted inferences regarding the last empirical evidence, we report in Table 6b (see column *HFSF*) a summary of the additional (i.e. taxpayers’ losses) or the lower (i.e. taxpayers’ gains) level of capital that the HFSF would need to inject into the country’s banking system compared to the level of capital that was actually raised in order to support the current formation of the four systemic banks and the current formation of the sector in improving its soundness in the aftermath of the financial turmoil. The results suggest the last specific M &A wave of both Alpha Bank and Ethniki Bank consists of the optimum selection of financial institutions that leads to the highest economic gains (see the negative HFSF values). On the contrary, Eurobank and Piraeus Bank could have been involved in a consolidation activity with alternative financial institutions (other than those they actually got involved with during the last M &A wave), which could have resulted in effective alleviation of the tax burden, as the level of the aforementioned recapitalization of each one of the four systemic banks from EFSF via the HFSF, could have been smaller. Our findings are in line with the inferences of Chong et al. (2006) and Granja et al. (2017). Specifically, the former study provides evidence on cronyism and argues that when the matching of merger partners is forced by the authorities it destroys economic value; while the second one demonstrates that misallocation of failed banks to potential acquirers results in significant economic losses.

#### The UK and Greece

Table 7 illustrates the average gains or losses that stem from all the prospective consolidation activity of the largest banks in each banking sector in both the pre- and post-crisis periods. As far as the UK banking sector is concerned, the results suggest that pre-crisis, all large banks’ potential combinations of M &A would generate gains for the UK economy, whereas in the aftermath of the crisis, this could only occur for the M &A cases of Barclays and HSBC. Regarding the Greek banking sector, the empirical evidence highlights a similar picture as the UK one. Specifically, in both eras around the crisis only two financial institutions of the so-called big four of the sector, namely Alpha Bank and Ethniki Bank, seem to create synergies that can result in cost reduction, while Eurobank and Piraeus Bank have a positive impact to the economy only in the pre-crisis period. This is a quite surprising finding with regards to the post-crisis period given the new systemic formation (as a result of the recent post-crisis consolidation wave) of those four banks and their emerging importance as the new cornerstones of the Greek economy.

**Table 7:** UK & Greece—Largest Banks’ M &As Scenarios Gain/Losses

	Pre Crisis (M)			Post Crisis (M)		
	Min	Max	Mean	Min	Max	Mean
Panel A: UK						
BARCLAYS	$-$ 4387.14	4846.46	4410.91	$-$ 17,697.3	9968.47	8835.41
HSBC	$-$ 3894.17	4057.69	3903.56	$-$ 5382.91	7358.25	7221.89
LlOYDS	$-$ 4802.56	5092.21	4929.48	$-$ 6944.92	1834.3	$-$ 6408.41
RBS	$-$ 8432.48	2879.84	514.63	$-$ 7921.57	1967.75	$-$ 6976.79
SANTANDER	$-$ 1272.17	1339.35	1155.13	$-$ 2159.51	1999.53	$-$ 1959.15
STANDARD	$-$ 1346.16	1462.72	1385.06	$-$ 6497.3	1693.62	$-$ 2441.2
Panel B: Greece						
ALPHA	$-$ 162.92	240.67	91.11	$-$ 194.55	352.63	46.08
EUROBANK	$-$ 28.44	151.72	40.26	$-$ 278.79	112.87	$-$ 110.48
ETHNIKI	29.99	157.17	104.28	$-$ 189.22	383.19	82.19
PIRAEUS	$-$ 157.86	183.91	19.25	$-$ 358.94	146.45	$-$ 118.49

This table presents both the range and the average of gains (positive sign) or of losses (negative sign) in real money values(for UK in $\pounds$ and for Greece in €), resulting from each hypothetical M &A of the largest banks (in terms of assets, deposits and loans) in each banking system that is quoted for both the ‘pre’ crisis and the ‘post’ crisis period. M’ stands for million

#### Strategic fit

We finally turn our focus to the strategic and organisational fit between the financial institutions that are involved in each consolidation activity. According to the Similarity Index (SI) column displayed in Table 6a and b, in both countries those M &As that generate cost efficiency surplus have smaller SI levels (i.e., smaller strategic differences) regardless of the technological class that they belong to. Though the empirical evidence highlights that the new financial institutions that are classified in the first and more efficient regime depict lower SI values compared to those that are found in the second technological regime. Our findings, are in line with Ramaswamy (1997) and Altunbas and Marques-Ibanez (2008) and suggest that the more strategically similar are the involved institutions in a consolidation activity the higher is the chance that their M &A will generate a cost efficiency surplus. Specifically, dissimilarities on earnings, capitalisation, loan and deposit strategies are the most important factors that contribute to the creation of cost efficiency losses.^37^ This supports the view that obstacles often arise when institutions with different strategic orientations integrate.

## Concluding remarks

In this paper, we propose an econometric method to evaluate and compare *ex*- *ante* the risk-adjusted efficiency gains or losses in real money terms of a potential (i.e., before it is realised) consolidation activity under different technological regimes. The performance of our approach is tested in the banking sector as it is the dominant sector of a country’s financial system. In this spirit, evidence is provided on the existence of heterogeneous technological classes in two different banking systems in terms of sophistication, market characteristics, and volume of transactions, those of the UK and Greece. Contrary to previous cross-country studies in the framework of an LCSFM model that derive their country-specific inferences by assuming a common sample for all different countries and thus neglecting substantial differences that exist among them, we attempt to compare the countries of interest by examining them separately. Furthermore, we employ two different modelling strategies to test the sensitivity and the robustness of our results. To the best of our knowledge from all previous efficiency-related banking studies, not only is the period we investigate the most extended, but we allow for different financial institutions in terms of their activities. The former allows us to account for all the important developments of both banking sectors, while the latter enables us to thoroughly examine the entire banking system of each country.

We provide detailed empirical evidence of an enhanced efficiency in both countries as well as important cost reductions as a result of prospective M &A that can be proved to be a significant factor in the alleviation of taxpayers’ burden. We show that similarities in strategic characteristics play a crucial role in the creation of post-consolidation cost efficiency surplus. Thus, in a circumspect manner, we cast doubt on the decisions of the policymakers with regard to the selection of specific acquirers and targets during the last wave of consolidation that took place in the Greek banking sector and on its ability to generate the most optimum synergies from an economic benefit point of view. Furthermore, the results suggest that bank heterogeneity in both countries is fully captured by two different technological classes. More precisely, the first regime in each banking system consists of the most efficient credit institutions. Finally, we present a trade-off with regards to efficiency and the level of sophistication of a banking system. The findings hold across both different modelling strategies that we follow and after various robustness tests that we perform.

All in all, this study presents important policy implications for the post COVID-19 era. The massive application of digital technologies during the current decade has favoured the entry of new FinTech firms, as well as BigTech players in banking-related activities and has intensified the competition with traditional bank business models in particular in the area of payments. This could lead to a wide restructuring of the banking sectors and could make consolidation the only way either to survive or to become competitive. Thus, efficiency gains, such as significant cost reductions, that can be derived from appropriate consolidation actions can enable economic prosperity and growth as well as to lead to tax alleviation especially in the case of a bailout scenario. With, this in mind, worldwide banking systems, in both the Economic and Monetary Union (EMU) area with the ongoing banking union process and other developed and emerging economies, should be empirically investigated as well. Finally yet importantly, our proposed methodology can be applied to any micro-study and provide social planners with an important tool to evaluate the optimal industrial organization of any economic sector. In turn, it would be interesting to explore different industries and sectors of the economy, in order to extract inferences with important policy implications on potential economic benefits that a consolidation activity may have, especially in stressed periods, such as the current ongoing economic uncertainty due to the COVID-19 pandemic.

## Appendix

### 1. Which technological regime is the most efficient?

Tables A1a and A1b report average cost efficiency estimates using the highest probability cost frontier as a reference technology with respect to the UK and Greece. It is revealed that for both countries the first technological regime consists of banks that exhibit higher cost efficiency levels compared to the second one. It is noteworthy to highlight that in 2007 for the UK and in 2008 for Greece, efficiency levels started to decline at the highest rate during both of the sample periods. This coincides with the dawn of the global financial crisis in August 2007 and the turmoil of the global money markets that followed and reached the point of eruption with the collapse of Lehman Brothers in September 2008. An overall comparison of all the banks in both banking systems for the entire common sample period (1993–2011) emphasizes the fact that Greek banks operate under higher efficiency levels than their European counterparties, albeit their systems are more sophisticated , a result that is in line with Casu and Girardone (2006). The answer to this conundrum could lie in the simplicity of activities and in the smaller size of the Greek banking sector. This is a point that has triggered various debates related to the diversity of banking activities and the complexity of financial systems (Arcand et al., 2012; Cecchetti and Kharoubi, 2012).

**Table 8:** (a) UK—Average cost efficiency estimates, (b) Greece—Average cost efficiency estimates

Overall sample			LCM			
			Class1		Class2	
Year	Mean	Obs.	Mean	Obs.	Mean	Obs.
(a)						
1988	0.68	6	0.68	6	–	–
1989	0.69	29	0.73	22	0.48	7
1990	0.68	38	0.71	28	0.46	10
1991	0.68	42	0.7	31	0.49	11
1992	0.67	50	0.71	37	0.47	13
1993	0.66	52	0.69	38	0.48	14
1994	0.65	53	0.7	39	0.47	14
1995	0.65	62	0.69	42	0.5	20
1996	0.66	85	0.71	56	0.41	29
1997	0.68	89	0.67	58	0.43	31
1998	0.7	89	0.73	57	0.42	32
1999	0.69	90	0.72	55	0.42	35
2000	0.66	92	0.71	56	0.41	36
2001	0.65	96	0.73	59	0.34	37
2002	0.64	100	0.71	58	0.35	42
2003	0.64	103	0.71	59	0.39	44
2004	0.65	103	0.72	58	0.41	45
2005	0.64	104	0.71	58	0.4	46
2006	0.64	103	0.71	56	0.4	47
2007	0.62	99	0.7	57	0.37	42
2008	0.62	98	0.69	56	0.36	42
2009	0.61	97	0.68	55	0.34	42
2010	0.59	94	0.66	53	0.32	41
2011	0.56	82	0.63	50	0.3	32
Total	0.65	1856	0.7	1144	0.41	712
(b)						
1993	0.69	21	0.77	13	0.44	8
1994	0.68	21	0.76	13	0.46	8
1995	0.69	21	0.77	13	0.49	8
1996	0.72	21	0.78	13	0.56	8
1997	0.76	21	0.8	13	0.52	8
1998	0.76	20	0.78	12	0.59	8
1999	0.73	16	0.76	8	0.57	8
2000	0.72	15	0.78	7	0.63	8
2001	0.73	16	0.78	7	0.65	9
2002	0.72	19	0.8	8	0.63	11
2003	0.71	20	0.8	9	0.64	11
2004	0.79	20	0.85	9	0.75	11
2005	0.82	20	0.88	9	0.76	11
2006	0.83	18	0.9	9	0.79	9
2007	0.86	18	0.91	9	0.82	9
2008	0.85	18	0.89	9	0.81	9
2009	0.84	18	0.89	9	0.81	9
2010	0.82	18	0.86	9	0.79	9
2011	0.79	15	0.83	8	0.77	7
Total	0.76	356	0.82	187	0.66	169

(a) Reports the average cost efficiency estimates for each year of the UK banking industry with respect to the number of financial institutions that belong to the first and to the second technological class

(b) Reports the average cost efficiency estimates for each year of the Greek banking industry with respect to the number of financial institutions that belong to the first and to the second technological class

Additionally, in Tables A1a and A1b, we can see essential differences for each year in the efficiency estimates within the two classes in both the UK and the Greek banking sectors. More precisely, the average level of efficiency in the first technological class for Greece is close to 82%, whereas in the second technological class it is close to $66\%$. The gap between the two regimes is even larger in the UK. Specifically, the overall efficiency of class one and class two is approximately $70\%$ and $41\%$, respectively. Therefore, we highlight that the first technological regime in both banking systems consists of financial intermediaries that exhibit, on aggregate, higher cost efficiency levels compared to those that belong to the second latent class.

### 2. Interpretation of heterogeneous technologies using the determinants

The parameter estimates of our LCSFM are presented in Tables A3a and A3b for the UK and Greece, respectively, and are estimated by maximum likelihood estimation using NLogit 5 (Greene, 2009). All the variables are normalized by their respective geometric mean. Thus, the translog form represents a second-order Taylor approximation around the geometric mean to any generic cost frontier. In both countries, the estimated cost frontier elasticities are found to be positive; in turn, the estimated cost frontiers are increasing in input prices and outputs. The signs of the parameter estimates of the variables included in the functional form suggest that the monotonicity and concavity properties are satisfied. In most cases, the estimated parameters of the efficiency frontiers are significant at the conventional confidence levels. From these two tables we note that in both technological regimes of the two different banking systems, the estimated *Lambda* $(\lambda _{k})$ parameter is statistically insignificant in contrast to a model that assumes homogeneous production technology. This suggests that bank heterogeneity is fully captured when a model with two classes is estimated.^38^

**Table 9:** (a) UK—Latent cost frontier, inefficiency and class determinants estimates, (b) Greece—Latent cost frontier, inefficiency and class determinants estimates

Technology Class	1		2	
	Coefficient	b/St.Er.	Coefficient	b/St.Er.
(a)				
*Kernel determinants*				
Constant	1.585	22.288	0.447	4.311
lnw1	0.059	7.732	0.071	2.495
lnw2	0.872	83.717	0.662	33.423
lny1	0.482	33.109	0.292	8.623
lny2	0.303	23.577	0.251	10.341
lny3	$-$ 0.031	$-$ 4.045	$-$ 0.039	$-$ 2.141
lnEC	0.183	9.549	0.321	4.681
lnNPLs	0.119	6.341	0.179	5.283
Trend	$-$ 0.001	$-$ 0.647	0.013	2.036
0.5(lnw1)$^{\wedge }$2	0.598	4.326	0.446	2.948
0.5(lnw2)$^{\wedge }$2	0.734	2.345	0.234	3.455
0.5(lny1)$^{\wedge }$2	0.937	4.743	0.829	4.809
0.5(lny2)$^{\wedge }$2	0.723	2.891	0.832	3.944
0.5(lny3)$^{\wedge }$2	$-$ 0.235	$-$ 3.549	$-$ 0.235	$-$ 4.283
0.5(Trend)$^{\wedge }$2	0.431	0.872	0.728	2.189
lnw1*lnw2	0.234	3.862	0.873	2.923
lny1*lny2	0.928	4.834	0.734	3.891
lny1*lny3	$-$ 0.927	$-$ 3.729	$-$ 0.892	$-$ 3.711
lny2*lny3	$-$ 0.823	$-$ 4.821	$-$ 0.824	$-$ 4.203
*Ineffficient determinants*				
TIME	$-$ 0.056	$-$ 5.589	0.047	3.153
SIZE	0.225	8.473	0.165	3.056
BS	$-$ 0.884	$-$ 2.207	0.007	0.059
*Class determinants*				
CONSTANT	0.78	5.944	Control Group	
CAPITAL ADEQUACY	0.568	6.056	Control Group	
LIQUIDITY RISK	$-$ 0.736	4.694	Control Group	
CREDIT RISK	$-$ 0.263	$-$ 4.513	Control Group	
SERV_CON	$-$ 0.628	$-$ 3.637	Control Group	
PROFITABILTY	1.472	0.864	Control Group	
Sigma	0.181	4.837	0.388	5.876
Lambda	0.358	0.608	0.307	1.044
Number of observations	1144		712	
Prior class probabilities at data means	0.573		0.427	
(b)				
*Kernel determinants*				
Constant	0.933	5.502	0.346	10.479
lnw1	0.042	6.286	0.713	12.876
lnw2	0.852	18.514	1.026	10.808
lny1	0.529	10.5	0.626	8.171
lny2	0.352	7.214	0.292	2.597
lny3	$-$ 0.017	$-$ 4.862	0.087	5.383
lnEC	0.133	3.034	0.023	4.156
lnNPLs	0.107	4.922	0.037	4.729
Trend	0.177	1.851	0.104	2.722
0.5(lnw1)$^{\wedge }$2	0.349	4.901	0.382	3.048
0.5(lnw2)$^{\wedge }$2	0.822	3.901	0.311	3.922
0.5(lny1)$^{\wedge }$2	0.725	2.893	0.915	3.171
0.5(lny2)$^{\wedge }$2	0.293	3.948	0.556	3.091
0.5(lny3)$^{\wedge }$2	$-$ 0.942	$-$ 3.955	$-$ 0.921	$-$ 2.872
0.5(Trend)$^{\wedge }$2	0.334	1.163	0.456	1.692
lnw1*lnw2	0.174	4.983	0.148	5.294
lny1*lny2	0.819	3.945	0.719	4.301
lny1*lny3	$-$ 0.117	$-$ 2.998	$-$ 0.632	$-$ 4.219
lny2*lny3	$-$ 0.487	$-$ 3.871	$-$ 0.718	$-$ 3.912
*Ineffficient determinants*				
TIME	$-$ 0.075	$-$ 3.244	$-$ 0.143	$-$ 3.969
SIZE	0.694	6.298	0.297	2.879
Owner	0.267	0.435	0.703	0.33
*Class determinants*				
CONSTANT	1.276	2.609	Control Group	
CAPITAL ADEQUACY	0.547	4.831	Control Group	
LIQUIDITY RISK	$-$ 0.947	$-$ 5.874	Control Group	
CREDIT RISK	$-$ 0.686	$-$ 3.039	Control Group	
SERV_CON	$-$ 0.097	$-$ 0.982	Control Group	
PROFITABILTY	0.001	0.222	Control Group	
Sigma	0.948	11.63	0.974	26.655
Lambda	0.118	0.422	0.24	0.402
Number of observations	187		169	
Prior class probabilities at data means	0.625		0.375	

(a) Feautures latent cost frontier, inefficiency, and class determinants estimates of 1856 observations for 124 UK financial institutions in the period 1988–2011. The estimation is conducted under a panel data nature methodology (Orea and Kumbhakar 2004) which allows the efficiency term to vary every year. Log likelihood is $-\,456.9226$. Lamda ($\lambda$) and Sigma ($\sigma$) are efficient parameters, where $\lambda (=\sigma u/\sigma v)$, the ratio of the standard deviation of efficiency over the standard deviation of the noise term, and $\sigma (=\sigma u+\sigma v)$, the composite standard deviation. The variables are as described in Table 2a

(b) Feautures latent cost frontier, inefficiency, and class determinants estimates of 356 observations for 30 Greek financial institutions in the period 1993–2011. The estimation is conducted under a panel data nature methodology (Orea and Kumbhakar 2004) which allows the efficiency term to vary every year. Log likelihood is 90.97407. Lamda ($\lambda )$ and Sigma ($\sigma$) are efficient parameters, where $\lambda (=\sigma u/\sigma v)$, the ratio of the standard deviation of efficiency over the standard deviation of the noise term, and $\sigma ( = \sigma u+\sigma v)$, the composite standard deviation. The variables are as described in Table 2b

Next, we examine the results that emanate from determinants that affect the inefficiency component. As far as the UK banks are concerned, we notice that in the first technological regime efficiency increases over time, whereas there is an erosion of efficiency throughout the years in the second one. This can be seen from the positive sign of the statistically significant determinant (i.e. *TIME*) in class two; inefficiency increases during the years of the sample. The significant and negative effect that *size* has on efficiency prevails in both classes, but there is a mixed effect of the nature of a financial institution in the two regimes. More precisely, the dummy variable *BS* does not have any significant effect in the second class; nonetheless, it has a detrimental effect on efficiency if the financial institution is a bank and not a building society.^39^

As far as the Greek banks are concerned, we notice similarities among the two different regimes in terms of the sign and the significance of the effect that *size* has on efficiency. Although we report similar results with respect to the banks that belong to the first technological regime in both countries’ banking sectors, the *time* determinant has exactly the opposite effect on efficiency in the Greek banking system (compared to the UK) regarding the banks that belong to the second technological regime. Last, we highlight that ownership has no important effect on the efficiency of banks, regardless of their classification among the two regimes.

Subsequently, we shed light on the differences of technology regimes based on the posterior production variable distributions. For both countries, the majority of determinants are statistically significant, indicating that they are critical for the classification of banks among the two regimes. Analysis of the class determinants in terms of their sign and their statistical significance suggests that in the UK banking sector the first technological regime is very likely to consist of banks with a strong capital base, with high-quality credit and liquidity risk management, and a broader scope in product provision. This outcome is in line with the main principles of the Basel accords^40^ regarding the adequate level of capital that each bank must hold on their balance sheets in order to become more efficient. On the contrary, banks not adequately capitalized, that undertake risky projects, and with parsimonious liquidity but increased service specialization are likely to be found in the second latent class. The effect of profitability is subdued.

Turning to the Greek banking sector, we notice that the banks that belong to those two different latent classes exhibit similar characteristics in terms of capital and the level of both credit and liquidity risk they undertake as the UK banks in the same regimes. The primary difference between the two classes and in essence between the two countries is that not only profitability but service concentration as well has a statistically insignificant effect on the classification process of the Greek banks (see Table A3b).

### 3. Classification of financial institutions

The classification of the banks into the two technological regimes is displayed in Tables A4a and A4b for the UK and Greek banking systems, respectively. The empirical evidence suggests that for both countries, each regime consists of institutions of similar characteristics, despite their differences in terms of the number of banks. This finding strengthens the motivation and scope of this paper, as it casts doubt on an a priori sample separation depending uniquely on banking segments. A thorough look into the two classes permits us to extract interesting inferences regarding the nature of the financial institutions that belong to each regime.

**Table 10:** (a) UK—Classification of banks, (b) Greece—Classification of banks

	Latent Class 1				Latent Class 2		
	Name	Years	Num OBS		Name	Years	Num OBS
(a)							
_1	ABC Int.	1996–2011	16	_1	AIB Group	1995–2011	17
_2	AIB Bank	1992–2008	17	_2	Abbey Nat.	1990–2011	22
_3	Adam & Company	1989–2011	23	_3	Alliance & Leic. Bank	1995–2006	12
_4	Ahli United	1989-2011	23	_4	Alpha Bank	1989–2011	23
_5	Alliance & Leic. Plc	1996–2011	16	_5	Anglo-Romanian	1989–2010	22
_6	Arbuthnot	1991–2011	21	_6	BMCE Int.	2006–2011	6
_7	Bank of China	2007–2011	5	_7	Bank Leumi	1996–2011	16
_8	Bank of Cyprus	1997–2003	7	_8	Bank Mandiri	1999–2011	13
_9	Bank of Tokyo	1988–1996	9	_9	Bank Saderat	1996–2011	16
_10	Barclays Bank	1992–2011	20	_10	Bank of Beirut	2002–2011	10
_11	Barclays Priv. & Tr.	2002–2005	4	_11	Bank of N.Y. Mellon	1997–2011	15
_12	Bath BS Sav. & Inv.	1995–2010	16	_12	Bank of Scotland	1990–2011	22
_13	Beneficial Bank	1988–1998	11	_13	Barclays Priv. Clien.	2002–2008	7
_14	Britannia BS	1989–2009	21	_14	Bradford & Bingley Bank	1999–2011	13
_15	Buckinghamshire BS	2003–2011	9	_15	British Arab	1989–2011	23
_16	Butterfield Guernsey	1996–2011	16	_16	Butterfield Holdings	1992–2010	19
_17	Cambridge BS	1996–2011	16	_17	Capital One	2002–2011	10
_18	Cheshire BS	1990–2007	18	_18	Chelsea BS	1990–2009	20
_19	Co-operative	1990–2011	22	_19	Citibank	1989–2011	23
_20	Coventry BS	1989–2011	23	_20	Cuscatlan Bank and Trust	2002–2006	5
_21	Credit Suisse	1997–2011	15	_21	DB UK	1996–2011	16
_22	Darlington BS	1996–2011	16	_22	Dunbar	1995–2010	16
_23	Dexia Municipal	1992–1999	8	_23	Egg	1996–2011	16
_24	Duncan Lawrie	2008–2010	3	_24	Europe Arab	2005–2011	7
_25	Dunfermline BS	1992–2007	16	_25	FBN	2003–2011	9
_26	FIBI	1996–2011	16	_26	Fairbairn	1998–2011	14
_27	Ghana	1998–2011	14	_27	Finsbury Pavement	1991–2006	16
_28	HSBC Middle East	1989–2011	23	_28	Gresham Trust	1993–2000	8
_29	HSBC	1989–2011	23	_29	HBOS	2000–2011	12
_30	Habib Allied	2001–2011	11	_30	Halifax	1996–2006	11
_31	Habibsons	1996–2011	16	_31	Heritable	1989–2007	19
_32	Isle of Man Bank Limited	1995–2011	17	_32	ICBC	2003–2011	9
_33	Italian Int.	1988–1997	10	_33	JP Morgan	1996–2011	16
_34	Kaupthing Singer & Friedlander	1989–2007	19	_34	Jordan Int.	1996–2011	16
_35	Kingdom	2009–2011	3	_35	KDB Bank	1992–1998	7
_36	Leeds BS	1989–2011	23	_36	Kookmin	1995–2010	16
_37	Lloyds (BLSA)	1992–2001	10	_37	Lazard & Co Holdings	1999–2011	13
_38	Lloyds	1988–1998	11	_38	London Int.	2001–2006	6
_39	Lloyds TSB	1998–2011	14	_39	MBNA Europe Bank	1995–2010	16
_40	Lloyds TSB Scotland	1989–2010	22	_40	Morgan Stanley	2001–2011	11
_41	London Trust	1991–1998	8	_41	Northern	1995–2010	16
_42	Manchester BS	1990–2011	22	_42	Northern Rock	1996–2011	16
_43	Marsden BS	1996–2011	16	_43	PNB	1997–2011	15
_44	Melli	2001–2011	11	_44	Progressive BS	1996–2011	16
_45	Melton Mowbray BS	1996–2011	16	_45	Riggs	1989–2004	16
_46	Merrill Lynch	1990–2005	16	_46	Sainsbury’s	2002–2011	10
_47	National Bank of Kuwait	1996–2011	16	_47	The Access	2008-2011	4
_48	National Counties BS	1996–2011	16	_48	Ulster	1989–2011	23
_49	National Westminster	1989–2011	23	_49	Union	2005–2011	7
_50	Nationwide BS	1990–2011	22	_50	United Trust	1999–2011	13
_51	Newcastle BS	1989–2011	23	_51	VTB Capital	2004–2011	8
_52	Nottingham BS	1992–2011	20				
_53	Principality BS	1989–2011	23				
_54	Prudential-Bache	1996–2001	6				
_55	Riyad	1993–1997	5				
_56	Royal Bank of Scotland Int.	1996–2008	13				
_57	Royal Bank of Scotland	1995–2011	17				
_58	Santander	1989–2011	23				
_59	Schroders	1989–2011	23				
_60	Secure Trust	1999–2011	13				
_61	Skipton BS	1989–2011	23				
_62	Standard	2000–2011	12				
_63	Standard Chartered	1998–2011	14				
_64	Standard Chartered Plc	1990–2011	22				
_65	Stroud & Swindon BS	1994–2009	16				
_66	Swansea BS	1996–2011	16				
_67	TSB	1988–1997	10				
_68	Turkish	1996–2011	16				
_69	United National	2001–2011	11				
_70	Unity Trust	1991–2011	21				
_71	Weatherbys	1997–2011	15				
_72	West Merchant	1988–1997	10				
_73	Yorkshire BS	1989–2011	23				
	Total		1144				712
(b)							
_1	Aegean Baltic	2003–2011	9	_1	Agricultural (ATE)	1993–2011	19
_2	Alpha	1993–2011	19	_2	Attica	1993–2011	19
_3	Bank of Athens	1993–1997	5	_3	Emporiki (Commercial)	1993–2011	19
_4	Bank of Central Greece	1993–1998	6	_4	FBB First Business	2002–2011	10
_5	Bank of Crete (Cretabank)	1993–1998	6	_5	General	1993–2011	19
_6	Ergobank	1993–1999	7	_6	Laiki	1993–2005	13
_7	Eurobank Ergasias	1993–2011	19	_7	Macedonia Thrace	1993–1999	7
_8	Ionian and Popular	1993–1998	6	_8	Marfin	1993–2005	13
_9	National Bank of Greece (Ethiki)	1993–2011	19	_9	Marfin Egnatia	1993–2010	18
_10	National Mortgage Bank	1993–1997	5	_10	Millennium	2000–2011	12
_11	PRObank	2001–2011	11	_11	Omega	2001–2004	4
_12	Pancretan Cooperative	2002–2011	10	_12	Panellinia	2005–2011	7
_13	Piraeus	1993–2011	19	_13	Proton	2002–2010	9
_14	T Bank	1993–2010	18				
_15	TELESIS Investment	1993–2000	8				
_16	TT Hellenic Postbank	1998–2011	14				
_17	Xiosbank	1993–1998	6				
	Total		187				169

(a) Reports the classification of 124 UK financial institutions for the period 1988–2011 into the two latent technological classes according to the regime membership determinants desribed in Table 2a

(b) Reports the classification of 30 UK financial institutions for the period 1993–2011 into the two latent technological classes according to the regime membership determinants desribed in Table 2b

Regarding the UK banking sector, the vast majority of the building societies appears to be in the first regime. Savings banks appear almost universally in the first regime as well. This implies that both building societies and savings banks exhibit rather high efficiency levels compared to commercial banks. One might conjecture that the miscellaneous activities of commercial banks may be the primary cause of financial turmoil like that we experienced starting in August 2007 and that inevitably had calamitous consequences for the economic growth of both developed and emerging markets. Thus, we provide evidence in favor of the separation between the investment and commercial arms of banks.^41^ To this end, some action has already taken place in the UK. Specifically, the Independent Commission on Banking (ICB) has proposed “ring fencing” retail and small business commercial banking from investment banking in the UK.^42^

Turning to the Greek banking sector, we find similar evidence to that described for the UK. Savings banks and one cooperative type of bank (Pancretan Cooperative) appear in the first technological regime; however, both regimes are actually dominated by commercial banks, as is the Greek banking sector in general. Nevertheless, the rest of the cooperative banks (such as Panellinia Bank) appear in the less efficient regime.^43^ As far as *ownership* is concerned, we highlight that it has rather a subdued effect, as there is an equal distribution of state-owned and privately owned banks between the two regimes. It’s noteworthy that most of the banks from the whole sample whose operations have been terminated either because they were acquired or because they were involved in a merging activity belong to the first technological group as well.

A common point to both countries is that the four largest banks (in terms of assets, deposits, and loans) are classified as being in the first regime. These are HSBC, RBS, Lloyds, and Barclays in the UK and Ethniki, Eurobank, Alpha, and Pireaus in Greece. This finding is of extreme importance for Greece, as the four aforementioned banks compose the four cornerstones of the recovery of the Greek economy.^44^ Consequently, the classification of all four systemic banks into the most efficient technological regime has major policy implications regarding the success and the scope of the last wave of banks’ M &A activity and in general for the country’s detachment from the recession after many consecutive years.

### 4. Robustness checks

In order to examine the robustness of our findings, we perform a series of robustness tests. First, as noted in section 3, we conduct exactly the same analysis, but instead of following Orea and Kumbhakar’s (2004) panel data methodology, we follow Bos et al.’s (2010) pooled cross-section strategy that allows the financial institution to be in one regime in a specific year and in another regime the year after. Unequivocally, for both countries the results do not reveal any significant differences regarding the number of different technological regimes (i.e. two classes) and the classification of banks among these two regimes (see Tables A5a and A5b). Specifically, more than $80\%$ of the yearly observations of each credit institution in both banking sectors are in the same class as they are when we use Orea and Kumbhakar’s (2004) modelling strategy. With respect to the remaining $20\%$, where for some year observations the credit institution seems to change class, we highlight that this transition occurs in no more than two consecutive years and in the first year observations for all the credit institutions that belong to this $20\%$ in both countries. The only rudimentary difference apparent in the results in both countries is that all the class membership determinants are statistically significant and larger than in the previous panel datasets. Consequently, we add to our previous findings that for both countries the credit institutions that belong to the first technological regime are more profitable compared with their peers in the second regime. Specifically, in terms of the Greek banking sector, it seems that the broader the variety of products the banks provide, the higher the probability for them to be classified in the first technological regime.^45^ This larger statistical significance is apparent in the case of the kernel and inefficiency determinants as well. It must be noted that no change in terms of signs is found. In turn, we argue that the influence of all determinants is in the same direction as before. Thus, we are confident regarding the correct number of identified distinct technological regimes, the appropriateness of our determinants to classify the credit institutions into the two regimes, and most importantly, the exact classification of each credit institution into one of the two technological groups.

**Table 11:** (a) UK—“Pooled-Cross Section Data”, Latent cost frontier, inefficiency and class determinants estimates, (b) Greece—“Pooled-Cross Section Data”, Latent cost frontier, inefficiency and class determinants estimates

Technology Class	1		2	
	Coefficient	b/St.Er.	Coefficient	b/St.Er.
(a)				
*Kernel determinants*				
Constant	1.782	24.642	0.732	5.249
lnw1	0.081	8.019	0.076	2.893
lnw2	0.928	92.761	0.676	23.884
lny1	0.491	36.534	0.292	8.623
lny2	0.303	23.577	0.428	15.093
lny3	$-$ 0.035	$-$ 4.824	$-$ 0.063	$-$ 3.691
lnEC	0.183	9.549	0.32	4.682
lnNPLs	0.117	7.547	0.262	5.911
Trend	0.009	4.37	0.054	2.847
0.5(lnw1)$^{\wedge }$2	0.451	5.924	0.842	5.032
0.5(lnw2)$^{\wedge }$2	0.519	2.944	0.834	6.234
0.5(lny1)$^{\wedge }$2	0.117	4.936	0.176	4.931
0.5(lny2)$^{\wedge }$2	0.817	4.922	0.092	4.931
0.5(lny3)$^{\wedge }$2	$-$ 0.931	$-$ 3.861	$-$ 0.174	$-$ 4.913
0.5(Trend)$^{\wedge }$2	0.921	3.031	0.294	3.914
lnw1*lnw2	0.898	4.041	0.918	3.149
lny1*lny2	0.947	5.107	0.625	4.834
lny1*lny3	$-$ 0.731	$-$ 4.293	$-$ 0.921	$-$ 3.863
lny2*lny3	$-$ 0.884	$-$ 5.084	$-$ 0.316	$-$ 5.938
*Ineffficient determinants*				
TIME	$-$ 0.295	$-$ 6.928	0.087	4.937
SIZE	0.971	8.931	0.329	3.982
BS	$-$ 0.739	$-$ 2.729	0.084	0.074
*Class determinants*				
CONSTANT	1.025	7.864	Control Group	
CAPITAL ADEQUACY	0.894	8.186	Control Group	
LIQUIDITY RISK	$-$ 0.942	5.138	Control Group	
CREDIT RISK	$-$ 0.648	$-$ 4.975	Control Group	
SERV_CON	$-$ 0.849	$-$ 4.013	Control Group	
PROFITABILTY	1.188	3.046	Control Group	
Sigma	0.236	7.317	0.658	11.914
Lambda	0.748	0.964	0.483	1.204
Number of observations	1144		712	
Prior class probabilities at data means	0.573		0.427	
(b)				
*Kernel determinants*				
Constant	1.024	5.749	0.412	11.723
lnw1	0.051	6.476	0.787	13.244
lnw2	0.938	19.247	1.122	11.625
lny1	0.604	11.264	0.714	8.668
lny2	0.378	7.461	0.313	2.934
lny3	$-$ 0.019	$-$ 4.903	0.091	5.427
lnEC	0.144	3.854	0.051	4.764
lnNPLs	0.102	4.661	0.048	3.837
Trend	0.204	2.314	0.187	2.876
0.5(lnw1)$^{\wedge }$2	0.529	4.941	0.994	3.943
0.5(lnw2)$^{\wedge }$2	0.934	4.019	0.194	4.864
0.5(lny1)$^{\wedge }$2	0.915	4.902	0.831	3.917
0.5(lny2)$^{\wedge }$2	0.918	3.974	0.955	3.951
0.5(lny3)$^{\wedge }$2	$-$ 0.942	$-$ 3.045	$-$ 0.877	$-$ 4.044
0.5(Trend)$^{\wedge }$2	0.392	2.981	0.827	3.941
lnw1*lnw2	0.553	4.952	0.941	4.941
lny1*lny2	0.866	4.916	0.963	4.933
lny1*lny3	$-$ 0.772	$-$ 3.017	$-$ 0.329	$-$ 4.926
lny2*lny3	$-$ 0.951	$-$ 3.954	$-$ 0.922	$-$ 4.021
*Ineffficient determinants*				
TIME	$-$ 0.128	$-$ 4.921	$-$ 0.208	$-$ 4.021
SIZE	0.974	5.922	0.819	2.926
Owner	0.766	1.261	0.641	0.887
*Class determinants*				
CONSTANT	1.258	2.897	Control Group	
CAPITAL ADEQUACY	0.639	4.924	Control Group	
LIQUIDITY RISK	$-$ 1.014	$-$ 6.013	Control Group	
CREDIT RISK	$-$ 0.816	$-$ 3.944	Control Group	
SERV_CON	$-$ 0.849	$-$ 2.975	Control Group	
PROFITABILTY	0.758	2.496	Control Group	
Sigma	0.988	13.47	1.013	27.486
Lambda	0.247	0.549	0.285	0.501
Number of observations	187		169	
Prior class probabilities at data means	0.642		0.358	

(a) Presents latent cost frontier, inefficiency, and class determinants estimates of 1856 observations for 124 UK financial institutions in the period 1988–2011. The estimation is conducted under a pooled cross-section methodology (Bos et al. 2010) which permits each financial institution to switch between technology regimes over time. Log likelihood is $-\,431.6557$. Lamda ($\lambda$) and Sigma ($\sigma$) are efficient parameters, where $\lambda (=\sigma u/\sigma v)$, the ratio of the standard deviation of efficiency over the standard deviation of the noise term, and $\sigma ( = \sigma u+\sigma v)$, the composite standard deviation. The variables are as described in Table 2a

(b) Presents latent cost frontier, inefficiency, and class determinants estimates of 356 observations for 30 Greek financial institutions in the period 1993–2011. The estimation is conducted under a pooled cross-section methodology (Bos et al. 2010) which permits each financial institution to switch between technology regimes over time. Log likelihood is 98.4726. Lamda ($\lambda$) and Sigma ($\sigma$) are efficient parameters, where $\lambda ( = \sigma u/\sigma v)$, the ratio of the standard deviation of efficiency over the standard deviation of the noise term, and $\sigma ( = \sigma u+\sigma v)$, the composite standard deviation. The variables are as described in Table 2b

Next, we notice that the level of loan to loss provisions increases considerably after 2007 and 2008 for the UK and Greece, respectively. Some concerns arise regarding the scenario that our results-in terms of efficiency and allocation of banks to the two technological regimes-may be biased as they may be driven by the global financial crisis. In order to exclude any element of the crisis and examine the heterogeneity of the two banking sectors in a tranquil period, we truncate our sample and re-estimate our model without including the period 2007–2011 for both countries. As far as the UK banks are concerned, we note in Table A6a that $20\%$ of the banks (10 banks) that belonged to the second (and less efficient) regime move to the first class, whereas less than $5\%$ of banks (three banks) move from the first to the second class.^46^

As far as the Greek banks are concerned, we notice that in Table A6b the classification remains almost unchanged.^47^ Thus, we have strong evidence that our inferences regarding the Greek banking sector are extracted with precision. Similar to the initial results (i.e. where we follow the panel-data estimation strategy; see section 4), we can conclude again that the financial crisis had a greater impact on the UK banking sector than in the Greek banking sector. Specifically, it had a severe impact on the technology of the UK financial institutions, which made them quite cost inefficient. Consequently, their initial position deteriorated and they have moved further away from the efficient frontier.^48^

**Table 12:** (a) UK—Classification of banks before the financial crisis, (b) Greece—Classification of banks before the financial crisis

	Latent Class 1				Latent Class 2		
	Name	Years	Num OBS		Name	Years	Num OBS
(a)							
_1	ABC Int.	1996–2006	11	_1	AIB Group	1995–2006	12
_2	AIB Bank	1992–2006	15	_2	Abbey Nat.	1990–2006	17
_3	Adam & Company	1989–2006	18	_3	Alliance & Leic. Bank	1995–2006	12
_4	Ahli United	1989–2006	18	_4	Alpha Bank	1989–2006	18
_5	Alliance & Leic. Plc	1996–2006	11	_5	Anglo-Romanian	1989–2006	18
_6	Arbuthnot	1991–2006	16	_6	Bank Leumi	1996–2006	11
_7	Bank of Cyprus	1997–2003	7	_7	Bank Mandiri	1999–2006	8
_8	Bank of Tokyo	1988–1996	9	_8	Bank Saderat	1996–2006	11
_9	Barclays Bank	1992–2006	15	_9	Bank of Beirut	2002–2006	5
_10	Barclays Priv. & Tr.	2002–2005	4	_10	Bank of N.Y. Mellon	1997–2006	10
_11	Bath BS Sav. & Inv.	1995–2006	12	_11	Barclays Priv. Clien.	2002–2006	5
_12	Beneficial Bank	1988–1998	11	_12	British Arab	1989–2006	18
_13	Britannia BS	1989–2006	18	_13	Butterfield Holdings	1992–2006	15
_14	Buckinghamshire BS	2003–2006	4	_14	Cuscatlan Bank and Trust	2002–2006	5
_15	Butterfield Guernsey	1996–2006	11	_15	DB UK	1996–2006	11
_16	Cambridge BS	1996–2006	11	_16	Dunbar	1995–2006	12
_17	Cheshire BS	1990–2006	17	_17	Egg	1996–2006	11
_18	Co-operative	1990–2006	17	_18	FBN	2003–2006	4
_19	Coventry BS	1989–2006	18	_19	Fairbairn	1998–2006	9
_20	Credit Suisse	1997–2006	10	_20	Finsbury Pavement	1991–2006	16
_21	Darlington BS	1996–2006	11	_21	Gresham Trust	1993–2000	8
_22	Dexia Municipal	1992–1999	8	_22	Halifax	1996–2006	11
_23	Dunfermline BS	1992–2006	15	_23	Heritable	1989–2006	18
_24	FIBI	1996–2006	11	_24	ICBC	2003–2006	4
_25	HSBC Middle East	1989–2006	18	_25	JP Morgan	1996–2006	11
_26	HSBC	1989–2006	18	_26	Jordan Int.	1996–2006	11
_27	Habib Allied	2001–2006	6	_27	KDB Bank	1992–1998	7
_28	Habibsons	1996–2006	11	_28	Kookmin	1995–2006	12
_29	Isle of Man Bank Limited	1995–2006	12	_29	Lazard & Co Holdings	1999–2006	8
_30	Italian Int.	1988–1997	10	_30	London Int.	2001–2006	6
_31	Kaupthing Singer & Friedlander	1989–2006	18	_31	Morgan Stanley	2001–2006	6
_32	Leeds BS	1989–2006	18	_32	PNB	1997–2006	10
_33	Lloyds (BLSA)	1992–2001	10	_33	Progressive BS	1996–2006	11
_34	Lloyds	1988–1998	11	_34	Riggs	1989–2004	16
_35	Lloyds TSB	1998–2006	9	_35	Sainsbury’s	2002–2006	5
_36	Lloyds TSB Scotland	1989–2006	17	_37	United Trust	1999–2006	8
_37	London Trust	1991–1998	8	_38	VTB Capital	2004–2006	3
_38	Manchester BS	1990–2006	17	_39	**Ghana**	1998–2006	9
_39	Marsden BS	1996–2006	11	_40	**Riyad**	1993–1997	5
_40	Melli	2001–2006	6	_41	**United National**	2001–2006	6
_41	Melton Mowbray BS	1996–2006	11				
_42	Merrill Lynch	1990–2005	16				
_43	National Bank of Kuwait	1996–2006	11				
_44	National Counties BS	1996–2006	11				
_45	National Westminster	1989–2006	17				
_46	Nationwide BS	1990–2006	17				
_47	Newcastle BS	1989–2006	18				
_48	Nottingham BS	1992–2006	15				
_49	Principality BS	1989–2006	18				
_50	Prudential-Bache	1996–2001	6				
_51	Royal Bank of Scotland Int.	1996–2006	11				
_52	Royal Bank of Scotland	1995–2006	12				
_53	Santander	1989–2006	18				
_54	Schroders	1989–2006	18				
_55	Secure Trust	1999–2006	8				
_56	Skipton BS	1989–2006	18				
_57	Standard	2000–2006	7				
_58	Standard Chartered	1998–2006	9				
_59	Standard Chartered Plc	1990–2006	17				
_60	Stroud & Swindon BS	1994–2006	13				
_61	Swansea BS	1996–2006	11				
_62	TSB	1988–1997	10				
_63	Turkish	1996–2006	11				
_64	Unity Trust	1991–2006	16				
_65	Weatherbys	1997–2006	10				
_66	West Merchant	1988–1997	10				
_67	Yorkshire BS	1989–2006	18				
_68	**Bank of Scotland**	1990–2006	17				
_69	**Bradford & Bingley Bank**	1999–2006	8				
_70	**Capital One**	2002–2006	5				
_71	**Chelsea BS**	1990–2006	17				
_72	**Citibank**	1989–2006	18				
_73	**HBOS**	2000–2006	7				
_74	**MBNA Europe Bank**	1995–2006	12				
_75	**Northern**	1995–2006	12				
_76	**Northern Rock**	1996–2006	11				
_77	**Ulster**	1989–2006	18				
	Total		980				403
(b)							
_1	Aegean Baltic	2003–2006	4	_1	Agricultural (ATE)	1993–2006	14
_2	Alpha	1993–2006	14	_2	Attica	1993–2006	14
_3	Bank of Athens	1993–1997	5	_3	Emporiki (Commercial)	1993–2006	14
_4	Bank of Central Greece	1993–1998	6	_4	FBB First Business	2002–2006	5
_5	Bank of Crete (Cretabank)	1993–1998	6	_5	General	1993–2006	14
_6	Ergobank	1993–1999	7	_6	Laiki	1993–2005	13
_7	Eurobank Ergasias	1993–2006	14	_7	Macedonia Thrace	1993–1999	7
_8	Ionian and Popular	1993–1998	6	_8	Marfin	1993–2005	13
_9	National Bank of Greece (Ethiki)	1993–2006	14	_9	Marfin Egnatia	1993–2006	14
_10	National Mortgage Bank	1993–1997	5	_10	Omega	2001–2004	4
_11	PRObank	2001–2006	6	_11	Proton	2002–2006	5
_12	Pancretan Cooperative	2002–2006	5				
_13	Piraeus	1993–2006	14				
_14	T Bank	1993–2006	14				
_15	TELESIS Investment	1993–2000	8				
_16	TT Hellenic Postbank	1998–2006	9				
_17	Xiosbank	1993–1998	6				
_18	**Millennium**	2000–2006	7				
	Total		150				117

(a) Reports the classification of 118 UK financial institutions for the period 1988–2006 (i.e. before the financial crisis) into the two latent technological classes according to the regime membership determinants desribed in Table 3a. Those financial insitutions that change class (compared with their previous classification where the sample was up to 2011 as diplayed in Table A4a) are labeled with a bold font

(b) Reports the classification of 29 Greek financial institutions for the period 1993–2006 (i.e. before the financial crisis) into the two latent technological classes according to the regime membership determinants desribed in Table 3b. Those financial insitutions that change class (compared with their previous classification where the sample was up to 2011 as diplayed in Table A4b) are labeled with a bold font

In order to be even more persistent in testing our implications regarding both the efficiency and the heterogeneity of the UK and Greek banks, we account for macroeconomic, financial, country-specific and bank-specific conditions, as previous studies have noted Pasiouras (2008). Therefore, we account for additional factors that we use both as inefficiency and class membership determinants. Regarding macroeconomic conditions, we take into consideration the level of real GDP growth. As far as financial traits are concerned, we account for the three-month treasury bill rate. Additionally, we account for a bank-specific financial factor, such as the stock return both in time *t* and $t-1$.^49^ We next consider specific dynamics regarding the nature of each banking sector. For this, we add to our analysis the Herfindahl Hirschman Index (HHI) to capture the concentration of each banking system and to examine whether it has any impact on the efficiency and consequently on technological heterogeneity among the banks. We calculate the HHI not only in terms of assets but in terms of loans and deposits as well so as to be as robust as possible. Furthermore, as in the case of the financial factors, we examine the bank-specific traits relating to the HHI. We account for the market power of each bank in the sample. Next, we consider the number of acquisitions the bank has made throughout the sample period, following a previous study that highlights the importance of this inclusion (Orea and Kumbhakar, 2004).^50^ Last, we account for the regulatory changes, following empirical evidence in the literature that suggests that who regulates (Central Bank or other authority) may have implications for efficiency (Gaganis and Pasiouras, 2013). With this in mind, as far as UK is concerned, we consider the fact that UK banks were regulated by the Financial Services Authority (FSA) between 2001-2013. Whereas for the case of Greece, we account for the country’s participation to the European Monetary Union (EMU) with the introduction of the Euro currency and the resulting independence of the Central Bank of Greece in 2001^51^. Unequivocally, for each country none of these determinants are found to be statistically significant, which could support their inclusion. This finding confirms our selection of determinants regarding their suitability in capturing and revealing all the differences in terms of efficiency and technological heterogeneity of the UK and Greek banking sectors.^52^

## Footnotes

1 Farrell & Shapiro (1990) demonstrate that prices will rise if a merger generates no synergies (efficiencies) and does not lower marginal costs. Thus, they argue that M &As can only contribute to social well-being when efficiency has increased substantially and when these gains are passed on to consumers.

2 A crucial criterion for judging potential mergers as acceptable is their ability to pass on merger-specific efficiency gains to customers. This efficiency pass-through criterion is explicitly stated in the US and EU merger guidelines (Neven, 2006; Hausman and Sidak, 2007; Werden, 2002) and is employed in Australia in an informal manner.

3 We highlight here, that our proposed methodology can also be applied to any micro-study not necessarily under a latent class context, such as industries and sectors where the participants (i.e., single or multiproduct firms) operate under homogenous production technologies.

4 Focarelli and Panetta (2003) highlight that, although in the short run consolidation generates adverse price changes, these are only temporary. Efficiency gains dominate the market power effect of mergers, leading to prices that are more favorable for consumers.

5 The similarity index was applied for the first time in the banking sector by Ramaswamy (1997), who examined the impact of strategic similarities between target and bidder firms on changes in postmerger performance in the U.S. banking industry.

6 This becomes clear if one looks at the $\pounds$ 550 billion UK government intervention following two bank rescue packages in 2008 and 2009 via the Special Liquidity Scheme and the Bank Recapitalisation Fund. Additionally, monetary authorities unavoidably had to take action and intervened by lowering interest rates. Finally, the Bank of England (BoE) committed $\pounds$ 375 billion to its asset purchasing program (quantitative easing) to date in order to give a further monetary stimulus to the economy.

7 In October 2008, the Greek government announced a €28 billion support package for Greek banks consisting of €5 billion worth of capital injections as far as a recapitalization scheme was concerned, €15 billion in state loan guarantees to credit institutions with varying maturity from three months up to three years in order for the banking system to meet its liquidity needs, and €8 billion worth of liquidity in the form of special bonds with maturity up to three years to be used as collateral to the Eurosystem and/or the interbank market for any credit provided by them.

8 Greece’s public debt amounted to about $390 billion by the end of the third term of 2009. $55 billion was held by Greek banks and the rest was held by foreign institutions, the majority of them European.

9 That led to the three bailout deals in May 2010 (€110 billion), in February 2012 (€130 billion) and in August 2015 (€86 billion) that were agreed between Greece and both the Eurozone countries and the International Monetary Fund (IMF).

10 CRD IV is an EU legislative package covering prudential rules for banks, building societies, and investment firms.

11 In addition see, Beard et al. (1997) and McNicholas and Murphy (2010) for studies which use a non-frontier approach.

12 We note that in the robustness section when we use the methodology of Bos et al. (2010), the conditional (on class *k*) log density in 8 is defined for each firm at each time *t* as in Greene (2002) where the inefficiency term varies freely over time (i.e., $u_{it\mid k}$ is *iid*). Thus, the overall contribution of firm i to the conditional likelihood in Greene is obtained as $LF_{ik}(\theta _{k})$ =$\prod \nolimits _{t=1}^{Ti}LF_{ik}(\theta _{k})$, where $LF_{ik}(\theta _{k})$ is the conditional likelihood function for firm *i* at time *t* and we treat each specific observation as independent throughout the years for each credit institution. In this way, we allow the financial institution to switch technological regimes in each year. Thus, the first methodology (Orea and Kumbhakar, 2004) adopts a panel-based approach, whereas the second (Bos et al., 2010) treats the data set as a pooled cross-section.

13 The reasoning behind selecting 1993 as the starting year for the sample regarding the Greek banking sector is because in that year, the Greek banking system was fully liberalized. This followed the provision of the Second Banking Directive regarding establishment, supervision, and operation in 1992 by the Basic Banking Law Banking Directive

14 Our sample consists of commercial banks, real estate and mortgage banks, bank holding companies, cooperative banks, and savings banks. Additionally, the sample period covers the years of the global financial crisis and the data reflect the fiscal interventions in both countries discussed in the introduction section.

15 Hughes et al. (2001) show that deposits are better modeled as inputs of production.

16 Numerous banks around the world have broadened their portfolio to offer non-traditional services. Additionally, OBS activities such as securitization, loan origination, derivative securities, and standby letters of credit among others have been expanding at a rapid pace. As a result, the share of fee-based and other non-interest income to total income has increased dramatically.

17 Berger and Mester (1997) argue that not accounting for equity can result in a scale bias, while the efficiency of banks could be miscalculated even if they behave optimally given their risk preferences.

18 The selection of both the dependent and independent variables is consistent with several other studies in the literature, (e.g. Berger and Mester 1997; Lozano-Vivas and Pasiouras 2010).

19 We highlight crucial points of our data selection strategy that have been omitted by the bulk of empirical studies that have used Bankscope database (Claessens and Horen, 2012; Clerides et al., 2015). This strategy is essential to ensuring the accuracy of results and inferences. First, both samples are checked for double-counted observations. Bankscope provides company account statements for banks and financial institutions worldwide, by collecting financial statements with both consolidated and unconsolidated statuses. Only the unconsolidated data are selected avoid double counting the same financial institution (in cases where unconsolidated data are not available, consolidated data were used). Additionally, M &As were taken into consideration, by thoroughly checking all M &A activities that took place within both banking sectors to ensure only the merged entity or the acquiring bank remained in the sample after take-over. For example, assuming that bank A and bank B merged in 2003 to create a new entity, bank C, then the two individual banks A and B are each included in the dataset until 2003. From 2003 onwards, these two banks’ operations are considered to be terminated and the new bank (bank C) is included in the database. In the same spirit, assume that bank A was acquired by bank B in 2003; both banks are included in the database until 2003, with bank A then becoming inactive after 2003 and bank B remaining active after 2003.

20 Our results are robust when we alternatively winsorize at 2.5% and 97.5% levels and at 1% and 99% levels.

21 We are aware that in the case of perfect competition, the bank with the lowest cost has the highest profit. Nevertheless, the HHI values shown in Table 1a and b, reveal that this is not the case in both banking sectors. This renders appropriate the choice of profitability as a class membership determinant.

22 We do not include the two dummy variables that we use to account for type (*bs*) and ownership (*owner*).

23 The translog function has been widely applied in the literature due to its flexibility. Berger and Mester (1997) found that both the translog and the Fourier-flexible form specifications yielded essentially the same average level and dispersion of measured efficiency, and both ranked the individual banks in almost the same order.

24 Normalization by equity capital makes sure that the banks are treated comparably and not subject to scale bias—otherwise the large banks would dominate the regressions (Berger and Mester, 1997).

25 In the case when more than two institutions are involved in a M &A, the SI formula considers the sum of the weighted average (according to each bank’s total assets) squared difference of each possible pair combination of banks.

26 We tried to estimate a model with more than two classes as well. In the case of the Greek banking sector, it failed to achieve convergence, indicating the model is over-specified. However, for the UK banking sector, neither multicollinearity nor over-specification prohibits convergence of the maximum likelihood estimator. That said, none of the parameters differ significantly from zero, and the number of observations in the additional regime is very small.

27 In Table 5 we provide detailed information regarding the formation of each one of the four systemic banks via the last M &A activity and their capital enhancement from the HFSF. We also report the remaining banks in the Greek banking sector.

28 In the first section in “Appendix” we show that for both countries the first technological regime consists of banks that exhibit higher cost efficiency levels compared to the second one.

29 To create the prospective M &A in both baking sectors, we follow Baltas et al.’s (2017) methodology. Specifically, the weighted sum for the main variables (*TC*, $y_{l=1,2,3}$, *EC*, *CR*), the inefficiency determinant (*SIZE*), and all the class membership determinants is computed for the banks involved in each potential M &A that we examine. With respect to the input prices ($w_{s=1,2,3}$), we compute the weighted average of the banks constituting each prospective M &A, while we treat the time trend (*T*) variable as before (i.e., $T=year1,year2...,final$-*year*). We select the first operating year in the sample that is common to all involved institutions as the starting year of each hypothetical consolidation scenario. For example, if bank A and B’s observations are between 1995 and 2011 and 2001 and 2011, respectively, then the hypothetical bank AB will be operating between 2001 and 2011. Consequently, the observations for both banks can be deleted within the overlapping period (i.e., 2001 to 2011). In the end, we re-estimate the cost efficiency of the new financial institution as it was explicitly described in Sect. 5 and specified by Eqs. 12–16.

30 The empirical evidence in both banking sectors reveals that the ’new’ financial institutions that are created after all prospective consolidation activity are classified in the two existing technological regimes, without creating a new technological class.

31 Regarding the recent M &A cases that the Greek banking sector experienced, we approach each one as a potential scenario in the economy, as our sample is dated up to 2011 and the last consolidation wave took place in 2012 and 2013. Additionally, to construct the potential M &A combinations we exclude the banks whose operations were terminated in the last year of our sample (i.e. 2011) and those who have terminated their operations anytime from 2011 to present to ensure that the results are of relative policy importance.

32 We have also considered each potential M &A case among the 9 financial institutions that belong ex-ante to the first class and we find the new bank to be classified in the same technological regime. The results are available upon request.

33 The UK government during the financial crisis 2007–2009 injected 20 and 45 Billion pounds (GB) in capital to Lloyds TSB and Royal Bank of Scotland respectively to support their soundness.

34 We perform a simple Chow (1960) test for a structural break at the beginning (i.e., in 2007) and in the end (i.e., in 2009) of the financial crisis and find strong evidence of a structural change in both points in time. In particular, the Chow test rejects the null hypothesis of no break (or constant parameter values), there by providing evidence that the difference in the subperiods regressions is statistically significant. Other studies, like that of Cornett et al. (2011), also use 2007 and 2009 as the starting and ending points of the crisis respectively.

35 We study every combination of potential M &A activity of Pireaus bank, which can consist of two to five banking institutions.

36 Attica bank and Aegean Bank are commercial banking institutions, whereas Panellinia Bank and Pancretan Co-operative Bank deal with co-operative banking activities.

37 For brevity purposes, we do not report the results of each strategic indicator for each M &A. The results are available upon request.
